# Supplementary material for: Design of modular autoproteolytic gene switches responsive to anti-coronavirus drug candidates
Source: Nat Commun. 2021 Nov 22;12:6786. doi: 10.1038/s41467-021-27072-3 (PMC8609006; doi:10.1038/s41467-021-27072-3)
Supplement: Supplementary file 1 — Supplementary Information [file 41467_2021_27072_MOESM1_ESM.pdf]

Supplementary Materials

Design of modular autoproteolytic gene switches responsive to anti-coronavirus drug candidates

Nik Franko[1], Ana Palma Teixeira[1], Shuai Xue[1], Ghislaine Charpin-El Hamri[2], Martin Fussenegger[1,3*]

[1] ETH Zurich, Department of Biosystems Science and Engineering, Mattenstrasse 26, CH-4058 Basel, Switzerland.

[2] Département Génie Biologique, Institut Universitaire de Technologie, Université Claude Bernard Lyon 1, F-69622, Villeurbanne Cedex, France

[3] University of Basel, Faculty of Life Science, Basel, Switzerland.

Correspondence: fussenegger@bsse.ethz.ch

Contents

1. **Supplementary Tables 1-7**
2. **Supplementary Figures 1-10**

Supplementary Table 1. PLpro cleavage sites

Cleavage site	Amino acid sequence
${}_{PLpro}CS_{NAT1}$ – (Nsp1/nsp2)	RELNGGAYTRYV
${}_{PLpro}CS_{NAT2}$ – (Nsp2/nsp3)	FTLKGGAPTKVT
${}_{PLpro}CS_{NAT3}$ – (Nsp3/nsp4)	IALKGGKIVNNW

Supplementary Table 2. Mpro cleavage sites

Cleavage site	Amino acid sequence
${}_{Mpro}CS_{NAT1}$ – (Nsp4/nsp5)	TSAVLQSGFRKM
${}_{Mpro}CS_{NAT2}$ – (Nsp5/nsp6)	SGVTFQSAVKRT
${}_{Mpro}CS_{NAT3}$ – (Nsp6/nsp7)	KVATVQSKMSDV
${}_{Mpro}CS_{NAT4}$ – (Nsp7/nsp8)	NRATLQAIASEF
${}_{Mpro}CS_{NAT5}$ – (Nsp8/nsp9)	SAVKLQNNELSP
${}_{Mpro}CS_{NAT6}$ – (Nsp9/nsp10)	ATVRLQAGNATE
${}_{Mpro}CS_{NAT7}$ – (Nsp10/nsp12)	REPMLQSADAQS
${}_{Mpro}CS_{NAT8}$ – (Nsp12/nsp13)	PHTVLQAVGACV
${}_{Mpro}CS_{NAT9}$ – (Nsp13/nsp14)	NVATLQAENVTG
${}_{Mpro}CS_{NAT10}$ – (Nsp14/nsp15)	TFTRLQSLENVA
${}_{Mpro}CS_{NAT11}$ – (Nsp15/nsp16)	FYPKLQSSQAWQ
${}_{Mpro}CS_{OPT}$	TTVRLQSGFRKM

Supplementary Table 3. Sequences of Mpro mutants from clinical samples

Mpro mutant	Amino acid sequence
**$Mpro_{C156F}$**	MSGFRKMAFPSGKVEGCMVQVTCGTTTLNGLWLDDVVYCPRHVICTSEDMLN<br>PNYEDLLIRKSNHNFLVQAGNVQLRVIGHSMQNCVLKLKVDTANPKTPKYKFV<br>RIQPGQTFSVLACYNGSPSGVYQCAMRPNFTIKGSFLNGSCGSVGFNIDYD**F**VSF<br>CYMHHMELPTGVHAGTDLEGNFYGPFVDRQTAQAAGTDTTITVNVLAWLYAA<br>VINGDRWFLNRFTTTLNDFNLVAMKYNYEPLTQDHVDILGPLSAQTGIAVLDM<br>CASLKELLQNGMNGRTILGSALLEDEFTPFDVVRQCSGVTFQ
**$Mpro_{G71S}$**	MSGFRKMAFPSGKVEGCMVQVTCGTTTLNGLWLDDVVYCPRHVICTSEDMLN<br>PNYEDLLIRKSNHNFLVQA**S**NVQLRVIGHSMQNCVLKLKVDTANPKTPKYKFV<br>RIQPGQTFSVLACYNGSPSGVYQCAMRPNFTIKGSFLNGSCGSVGFNIDYDCVSF<br>CYMHHMELPTGVHAGTDLEGNFYGPFVDRQTAQAAGTDTTITVNVLAWLYAA<br>VINGDRWFLNRFTTTLNDFNLVAMKYNYEPLTQDHVDILGPLSAQTGIAVLDM<br>CASLKELLQNGMNGRTILGSALLEDEFTPFDVVRQCSGVTFQ
**$Mpro_{R279C}$**	MSGFRKMAFPSGKVEGCMVQVTCGTTTLNGLWLDDVVYCPRHVICTSEDMLN<br>PNYEDLLIRKSNHNFLVQAGNVQLRVIGHSMQNCVLKLKVDTANPKTPKYKFV<br>RIQPGQTFSVLACYNGSPSGVYQCAMRPNFTIKGSFLNGSCGSVGFNIDYDCVSF<br>CYMHHMELPTGVHAGTDLEGNFYGPFVDRQTAQAAGTDTTITVNVLAWLYAA<br>VINGDRWFLNRFTTTLNDFNLVAMKYNYEPLTQDHVDILGPLSAQTGIAVLDM<br>CASLKELLQNGMNG**C**TILGSALLEDEFTPFDVVRQCSGVTFQ

**Mpro$_{V77A/K90R}$**	MSGFRKMAFPSGKVEGCMVQVTCGTTTLNGLWLDDVVYCPRHVICTSEDMLN<br>PNYEDLLIRKSNHNFLVQAGNVQLR**A**IGHSMQNCVLKL**R**VDTANPKTPKYKFV<br>RIQPGQTFSVLACYNGSPSGVYQCAMRPNFTIKGSFLNGSCGSVGFNIDYDCVSF<br>CYMHHMELPTGVHAGTDLEGNFYGPFVDRQTAQAAGTDTTITVNVLAWLYAA<br>VINGDRWFLNRFTTTLNDFNLVAMKYNYEPLTQDHVDILGPLSAQTGIAVLDM<br>CASLKELLQNGMNGRTILGSALLEDEFTPFDVVRQCSGVTFQ
**Mpro$_{P184L}$**	MSGFRKMAFPSGKVEGCMVQVTCGTTTLNGLWLDDVVYCPRHVICTSEDMLN<br>PNYEDLLIRKSNHNFLVQAGNVQLRVIGHSMQNCVLKLKVDTANPKTPKYKFV<br>RIQPGQTFSVLACYNGSPSGVYQCAMRPNFTIKGSFLNGSCGSVGFNIDYDCVSF<br>CYMHHMELPTGVHAGTDLEGNFYG**L**FVDRQTAQAAGTDTTITVNVLAWLYAA<br>VINGDRWFLNRFTTTLNDFNLVAMKYNYEPLTQDHVDILGPLSAQTGIAVLDM<br>CASLKELLQNGMNGRTILGSALLEDEFTPFDVVRQCSGVTFQ

Supplementary Table 4. Plasmids used and designed in this study

Plasmid	Detailed description and cloning strategy	Reference
pTS1017	O$_{TetR}$-P$_{hCMVmin}$_SEAP-pA<br><br>Mammalian reporter plasmid encoding O$_{TetR}$-P$_{hCMVmin}$ driven expression of SEAP reporter protein.	[1]
pLeo665	O$_{TetR}$-P$_{hCMVmin}$_ss-nanoLuc-pA<br><br>Mammalian reporter plasmid encoding O$_{TetR}$-P$_{hCMVmin}$ driven expression of secreted nanoLuc reporter protein.	[2]
pDA326	P$_{TREBI}$_Citrine-2A-SEAP-sTRSV-pA<br><br>Mammalian reporter plasmid encoding for P$_{TREBI}$-driven Citrine-2A-SEAP expression cassette with an sTRSV ribozyme-dependent destabilization module in the 3'-UTR.	[3]
BB3-YPet	P$_{hCMV}$_YPet-pA<br><br>Mammalian expression plasmid encoding P$_{hCMV}$-driven YPet expression.	[4]
BB3-5xUAS-SEAP	P$_{5xUAS}$_SEAP-pA<br><br>Mammalian reporter plasmid encoding P$_{5xUAS}$ driven expression of SEAP reporter protein.	[4]
pQP-T2A	Plasmid containing DNA binding domain GAL4.	Addgene plasmid #102583
pTS1106	P$_{hCMV}$_TetR-pA<br><br>Mammalian expression plasmid encoding P$_{hCMV}$-driven DNA binding domain TetR expression.	[4]
BB3-VP16	P$_{hCMV}$_VP16-pA<br><br>Mammalian expression plasmid encoding P$_{hCMV}$-driven VP16 expression.	[4]
BB3-rTetR	P$_{hCMV}$_rTetR-pA<br><br>Mammalian expression plasmid encoding P$_{hCMV}$-driven rTetR expression.	[4]
pAB150	P$_{hCMV}$_3xNLS-DcuR-VPR-pA	[1]

	Mammalian expression plasmid encoding P_{hCMV}-driven DcuR fused to a 3xNLS N-terminal domain and to a C-terminal VPR transactivator domain.	
BB3-P_{PGK}	P_{PGK}_MCS-pA<br><br>Mammalian expression plasmid encoding P_{PGK} and multi cloning site (MCS).	[4]
pNF024	P_{hCMV}_SEAP-pA<br><br>Mammalian expression plasmid encoding P_{hCMV}-driven SEAP reporter protein.	Unpublished
pcDNA3.1(+)	P_{hCMV}_MCS-pA<br><br>Cloning vector for constitutive expression of target genes	ThermoFisher
pDF145	P_{T7}_SpAH-Env140ac<br><br>*In vitro* RNA production plasmid without mammalian promoter activity.	[5]
rTetR-NS3-VP65_IRES_mCherry	Plasmid containing HCV protease.	Addgene plasmid #112628
pCDNA3-FlipGFP(TEV cleavage seq) t2A mCherry	Plasmid containing FlipGFP.	Addgene plasmid #124429
pNF118	P_{hCMV}_TetR-MproCS(opt)-NLS-VP16-pA<br><br>Mammalian expression plasmid encoding DNA binding domain TetR fused to transcription activation domain VP16 with fusion linker containing optimized Mpro cleavage site and NLS. TetR was amplified from pTS1106 using oNF249 and oNF251 to introduce optimised Mpro cleavage site, PCR fragment was digested with EcoRI/BamHI. VP16 was amplified from BB3-VP16 using oNF11 and oNF248 to introduce NLS, PCR fragment was digested with BamHI/XbaI. Fragments were ligated into pcDNA3.1(+) (EcoRI/XbaI).	This work
pNF120	P_{hCMV}_TetR-$_{PLpro}$CS$_{NAT3}$-NLS-VP16-pA<br><br>Mammalian expression plasmid encoding DNA binding domain TetR fused to transcription activation domain VP16 with fusion linker containing PLpro$_{NAT3}$ cleavage site and NLS. TetR was amplified from pTS1106 using oNF249 and oNF253 to introduce PLpro$_{NAT3}$ cleavage site, PCR fragment was digested with EcoRI/BamHI. VP16 was amplified from BB3-VP16 using oNF11 and oNF248 to introduce NLS, PCR fragment was digested with BamHI/XbaI. Fragments were ligated into pcDNA3.1(+) (EcoRI/XbaI).	This work
pNF121	P_{hCMV}_Mpro(S2)-15gs-TetR-$_{Mpro}$CS$_{OPT}$-NLS-VP16-pA<br><br>Mammalian expression plasmid encoding SARS-CoV-2 Mpro linked to N-terminal of DNA binding domain TetR fused to transcription activation domain VP16 with fusion linker containing Mpro$_{OPT}$ cleavage site and NLS. Mpro(S2) was amplified from synthetic gene fragment DNA_twist_Mpro(S2) using oNF258 and oNF259. TetR was amplified from pTS1106 using oNF260 and oNF251 to introduce Mpro$_{OPT}$ cleavage site. PCR assembly reaction using oNF258 and oNF251 was used to fuse Mpro(S2) and TetR-$_{Mpro}$CS$_{OPT}$ with 15gs linker, PCR fragment was digested with EcoRI/BamHI. VP16 was amplified from BB3-VP16 using oNF11 and oNF248 to introduce NLS, PCR fragment was digested with BamHI/XbaI. Fragments were ligated into pcDNA3.1(+) (EcoRI/XbaI).	This work
pNF122	P_{hCMV}_Mpro(S2)-15gs-TetR-VP16-pA	This work

	Mammalian expression plasmid encoding SARS-CoV-2 Mpro linked to N-terminal of DNA binding domain TetR fused to transcription activation domain VP16 with fusion linker containing NLS. Mpro(S2) was amplified from synthetic gene fragment DNA_twist_Mpro(S2) using oNF258 and oNF259. TetR was amplified from pTS1106 using oNF260 and oNF261. PCR assembly reaction, using oNF258 and oNF261 was used to fuse Mpro(S2) and TetR with 15gs linker, PCR fragment was digested with EcoRI/BamHI. VP16 was amplified from BB3-VP16 using oNF11 and oNF248 to introduce NLS, PCR fragment was digested with BamHI/XbaI. Fragments were ligated into pcDNA3.1(+) (EcoRI/XbaI).	
pNF123	P_{hCMV}_Mpro(S2)-pA<br><br>Mammalian expression plasmid encoding SARS-CoV-2 Mpro. Mpro(S2) was amplified from synthetic gene fragment DNA_twist_Mpro(S2) using oNF258 and oNF262, PCR fragment was digested with EcoRI/XbaI and ligated into pcDNA3.1(+) (EcoRI/XbaI).	This work
pNF124	P_{hCMV}_PLpro(S2)-pA<br><br>Mammalian expression plasmid encoding SARS-CoV-2 PLpro. PLpro(S2) was amplified from synthetic gene fragment DNA_twist_PLpro(S2) using oNF263 and oNF264, PCR fragment was digested with EcoRI/XbaI and ligated into pcDNA3.1(+) (EcoRI/XbaI).	This work
pNF138	P_{PGK}_TetR-$_{Mpro}CS_{OPT}$-NLS-VP16-pA<br><br>Mammalian expression plasmid encoding DNA binding domain TetR fused to transcription activation domain VP16 with fusion linker containing optimized Mpro cleavage site and NLS. P_{PGK} was excised from bb3-P_{PGK} using MluI/EcoRI and ligated into pNF118 (MluI/EcoRI).	This work
pNF140	P_{PGK}_Mpro(S2)-pA<br><br>Mammalian expression plasmid encoding SARS-CoV-2 Mpro. P_{PGK} was excised from bb3-P_{PGK} using MluI/EcoRI and ligated into pNF123 (MluI/EcoRI).	This work
pNF142	P_{PGK}_PLpro(S2)-pA<br><br>Mammalian expression plasmid encoding SARS-CoV-2 Mpro. P_{PGK} was excised from bb3-P_{PGK} using MluI/EcoRI and ligated into pNF124 (MluI/EcoRI).	This work
pNF143	P_{PGK}_Mpro(S2)-15gs-TetR-$_{Mpro}CS_{OPT}$-NLS-VP16-pA<br><br>Mammalian expression plasmid encoding SARS-CoV-2 Mpro linked to N-terminal of DNA binding domain TetR fused to transcription activation domain VP16 with fusion linker containing $Mpro_{OPT}$ cleavage site and NLS. P_{PGK} was excised from bb3-P_{PGK} using MluI/EcoRI and ligated into pNF121 (MluI/EcoRI).	This work
pNF144	P_{PGK}_Mpro(S2)-15gs-TetR-NLS-VP16-pA<br><br>Mammalian expression plasmid encoding SARS-CoV-2 Mpro linked to N-terminal of DNA binding domain TetR fused to transcription activation domain VP16 with fusion linker containing NLS. P_{PGK} was excised from BB3-P_{PGK} using MluI/EcoRI and ligated into pNF122 (MluI/EcoRI).	This work
pNF147	P_{PGK}_PLpro(S2)-15gs-TetR-$_{PLpro}CS_{NAT1}$-NLS-VP16-pA<br><br>Mammalian expression plasmid encoding SARS-CoV-2 PLpro linked to N-terminal of DNA binding domain TetR fused to transcription activation domain VP16 with fusion linker containing $PLpro_{NAT1}$ cleavage site and NLS. PLpro(S2) was amplified from synthetic gene fragment DNA_twist_PLpro(S2) using oNF263 and oNF272. TetR was amplified from pTS1106 using oNF260 and oNF252 to introduce	This work

	$PLpro_{NAT1}$ cleavage site. PCR assembly reaction using oNF263 and oNF252 was used to fuse PLpro(S2) and TetR-$_{PLpro}CS_{NAT1}$ with 15gs linker, PCR fragment was digested with EcoRI/BamHI. VP16 was amplified from BB3-VP16 using oNF11 and oNF248 to introduce NLS, PCR fragment was digested with BamHI/XbaI. Fragments were ligated into pNF140 (EcoRI/XbaI).	
pNF148	P_{PGK}_PLpro(S2)-15gs-TetR-NLS-VP16-pA<br><br>Mammalian expression plasmid encoding SARS-CoV-2 PLpro linked to N-terminal of DNA binding domain TetR fused to transcription activation domain VP16 with fusion linker containing NLS. PLpro(S2) was amplified from synthetic gene fragment DNA_twist_PLpro(S2) using oNF263 and oNF272. TetR was amplified from pTS1106 using oNF260 and oNF261 to introduce PLpro(nat1) cleavage site. PCR assembly reaction using oNF263 and oNF261 was used to fuse PLpro(S2) and TetR with 15gs linker, PCR fragment was digested with EcoRI/BamHI. VP16 was amplified from BB3-VP16 using oNF11 and oNF248 to introduce NLS, PCR fragment was digested with BamHI/XbaI. Fragments were ligated into pNF140 (EcoRI/XbaI).	This work
pNF151	$_{Otet}P_{hCMVmin}$_ss-nanoLuc_sTRSV-pA<br><br>Mammalian reporter plasmid encoding TetR driven expression of nanoLuc with a sTRSV ribozyme-dependent destabilization module in the 3'-UTR. nanoLuc was excised from pLeo665 using EcoRI/XbaI and ligated into pDA326 (EcoRI/XbaI).	This work<br><br><br>GenBank: OK425853
pNF152	P_{PGK}_TetR-15gs-$_{PLpro}CS_{NAT3}$-NLS-VP16-pA<br><br>Mammalian expression plasmid encoding DNA binding domain TetR fused to transcription activation domain VP16 with fusion linker containing $PLpro_{NAT3}$ cleavage site and NLS. P_{PGK} was excised from BB3-P_{PGK} using MluI and EcoRI and ligated into pNF120 (MluI/EcoRI).	This work
pNF153	P_{PGK}_Mpro(S2)-15gs-TetR-$_{Mpro}CS_{NAT1}$-NLS-VP16-pA<br><br>Mammalian expression plasmid encoding SARS-CoV-2 Mpro linked to N-terminal of DNA binding domain TetR fused to transcription activation domain VP16 with fusion linker containing Mpro(nat1) cleavage site and NLS. Mpro(S2) was amplified from synthetic gene fragment DNA_twist_Mpro(S2) using oNF258 and oNF259. TetR was amplified from pTS1106 using oNF260 and oNF250 to introduce $Mpro_{NAT1}$ cleavage site. PCR assembly reaction using oNF258 and oNF250 was used to fuse Mpro(S2) and TetR-MproCS(nat1) with 15gs linker, PCR fragment was digested with EcoRI/BamHI. VP16 was amplified from BB3-VP16 using oNF11 and oNF248 to introduce NLS, PCR fragment was digested with BamHI/XbaI. Fragments were ligated into pNF140 (EcoRI/XbaI).	This work
pNF157	P_{CMV}_Mpro(S2)-15gs-TetR-$_{Mpro}CS_{OPT}$-NLS-VPR-pA<br><br>Mammalian expression plasmid encoding SARS-CoV-2 Mpro linked to N-terminal of DNA binding domain TetR fused to transcription activation domain VPR with fusion linker containing $Mpro_{OPT}$ cleavage site and NLS. Mpro(S2)-TetR-$_{Mpro}CS_{OPT}$ was amplified from pNF143 using oNF258 and oNF304. VPR was amplified from pAB150 using oNF303 and oNF305. PCR assembly reaction using oNF258 and oNF305 to obtain Mpro(S2)-TetR-$_{Mpro}CS_{OPT}$-NLS-VPR, PCR fragment was digested with EcoRI/XhoI and ligated into pNF024 (EcoRI/XhoI).	This work
pNF159	P_{PGK}_Mpro(S2)-15gs-TetR-$_{Mpro}CS_{OPT}$-NLS-VPR-pA<br><br>Mammalian expression plasmid encoding SARS-CoV-2 Mpro linked to N-terminal of DNA binding domain TetR fused to transcription activation domain VPR with fusion linker containing $Mpro_{OPT}$ cleavage	This work

	site and NLS. P_{PGK} was excised from bb3-P_{PGK} using MluI/EcoRI and ligated into pNF157 (MluI/EcoRI).	
pNF162	P_{PGK}_PLpro(S2)-15gs-TetR-$_{PLpro}CS_{NAT3}$-NLS-VP16-pA<br><br>Mammalian expression plasmid encoding SARS-CoV-2 PLpro linked to N-terminal of DNA binding domain TetR fused to transcription activation domain VP16 with fusion linker containing $PLpro_{NAT3}$ cleavage site and NLS. PLpro(S2) was amplified from synthetic gene fragment DNA_twist_PLpro(S2) using oNF263 and oNF272. TetR-$_{PLpro}CS_{NAT3}$-NLS-VP16 was amplified from pNF120 using oNF260 and oNF248. PCR assembly reaction using oNF263 and oNF248 was used to fuse PLpro(S2) and TetR-$_{PLpro}CS_{NAT3}$-NLS-VP16, PCR fragment was digested with EcoRI/XbaI. Fragments were ligated into pNF140 (EcoRI/XbaI).	This work<br><br>GenBank: OK425852
pNF167	P_{PGK}_Mpro(S2)-15gs-TetR-$_{Mpro}CS_{OPT}$-NLS-VP64-pA<br><br>Mammalian expression plasmid encoding SARS-CoV-2 Mpro linked to N-terminal of DNA binding domain TetR fused to transcription activation domain VP64 with fusion linker containing $Mpro_{OPT}$ cleavage site and NLS. VP64 was amplified from pAB150 using oNF327 and oNF328 to introduce NLS, PCR fragment was digested with BamHI/XbaI and ligated into pNF143 (BamHI/XbaI).	This work<br><br>GenBank: OK425851
pNF168	P_{PGK}_Mpro(S2)-15gs-TetR-NLS-VP64-pA<br><br>Mammalian expression plasmid encoding SARS-CoV-2 Mpro linked to N-terminal of DNA binding domain TetR fused to transcription activation domain VP64 with fusion linker containing NLS. VP64 was amplified from pAB150 using oNF327 and oNF328 to introduce NLS, PCR fragment was digested with BamHI/XbaI and ligated into pNF144 (BamHI/XbaI).	This work
pNF169	P_{PGK}_TetR-NLS-VP16-pA<br><br>Mammalian expression plasmid encoding DNA binding domain TetR fused to transcription activation domain VP16 with fusion linker containing NLS. TetR was amplified from pTS1106 using oNF249 and oNF261, PCR fragment was digested with EcoRI/BamHI. VP16 was amplified from BB3-VP16 using oNF11 and oNF248 to introduce NLS, PCR fragment was digested with BamHI/XbaI. Fragments were ligated into pNF140 (EcoRI/XbaI).	This work
pNF174	P_{PGK}_Mpro(S1)-15gs-TetR-NLS-VP64-pA<br><br>Mammalian expression plasmid encoding SARS-CoV-1 Mpro linked to N-terminal of DNA binding domain TetR fused to transcription activation domain VP64 with fusion linker containing NLS. Mpro(S1) was amplified from synthetic gene fragment DNA_twist_Mpro(S1) using oNF332 and oNF333. TetR was amplified from pTS1106 using oNF260 and oNF261. PCR assembly reaction using oNF332 and oNF261 was used to fuse Mpro(S1) and TetR with 15gs linker, PCR fragment was digested with EcoRI/BamHI and ligated into pNF167 (EcoRI/BamHI).	This work
pNF175	P_{PGK}_PLpro(S1)-15gs-TetR-NLS-VP16-pA<br><br>Mammalian expression plasmid encoding SARS-CoV-1 PLpro linked to N-terminal of DNA binding domain TetR fused to transcription activation domain VP16 with fusion linker containing NLS. PLpro(S1) was amplified from synthetic gene fragment DNA_twist_PLpro(S1) using oNF334 and oNF335. TetR was amplified from pTS1106 using oNF260 and oNF261. PCR assembly reaction using oNF334 and oNF261 was used to fuse PLpro(S1) and TetR with 15gs linker, PCR fragment was digested with EcoRI/BamHI and ligated into pNF138 (EcoRI/BamHI).	This work

pNF179	P_{PGK}_Mpro(S1)-15gs-TetR-$_{Mpro}CS_{OPT}$-NLS-VP64-pA<br><br>Mammalian expression plasmid encoding SARS-CoV-1 Mpro linked to N-terminal of DNA binding domain TetR fused to transcription activation domain VP64 with fusion linker containing $Mpro_{OPT}$ cleavage site and NLS. Mpro(S1)-15gs-TetR-$_{Mpro}CS_{OPT}$ was amplified from pNF174 using oNF332 and oNF251, PCR fragment was digested with EcoRI/BamHI and ligated into pNF167 (EcoRI/BamHI).	This work
pNF180	P_{PGK}_PLpro(S1)-15gs-TetR-$_{PLpro}CS_{NAT3}$-NLS-VP16-pA<br><br>Mammalian expression plasmid encoding SARS-CoV-1 PLpro linked to N-terminal of DNA binding domain TetR fused to transcription activation domain VP16 with fusion linker containing $PLpro_{NAT3}$ cleavage site and NLS. PLpro(S1)-15gs-TetR-$_{PLpro}CS_{NAT3}$ was amplified from pNF175 using oNF334 and oNF253, PCR fragment was digested with EcoRI/BamHI and ligated into pNF138 (EcoRI/BamHI).	This work
pNF186	P_{PGK}_Mpro(S2)-15gs-GAL4BD-$_{Mpro}CS_{OPT}$-NLS-VP64-pA<br><br>Mammalian expression plasmid encoding SARS-CoV-2 Mpro linked to N-terminal of DNA binding domain GAL4 fused to transcription activation domain VP64 with fusion linker containing $Mpro_{OPT}$ cleavage site and NLS. Mpro(S2) was amplified from synthetic gene fragment DNA_twist_Mpro(S2) using oNF258 and oNF259. GAL4BD was amplified from pQP-T2A using oNF312 and oNF340 to introduce $Mpro_{OPT}$ cleavage site. PCR assembly reaction using oNF258 and oNF340 was used to fuse Mpro(S2) and GAL4BD-MproCS(opt) with 15gs linker, PCR fragment was digested with EcoRI/BamHI and ligated into pNF167 (EcoRI/ BamHI).	This work
pNF182	P_{PGK}_Mpro(S2)-15gs-GAL4BD-NLS-VP64-pA<br><br>Mammalian expression plasmid encoding SARS-CoV-2 Mpro linked to N-terminal of DNA binding domain GAL4 fused to transcription activation domain VP64 with fusion linker containing NLS. Mpro(S2) was amplified from synthetic gene fragment DNA_twist_Mpro(S2) using oNF258 and oNF259. GAL4BD was amplified from pQP-T2A using oNF312 and oNF326. PCR assembly reaction using oNF258 and oNF326 was used to fuse Mpro(S2) and GAL4BD with 15gs linker, PCR fragment was digested with EcoRI/BamHI and ligated into pNF167 (EcoRI/ BamHI).	This work
pNF184	P_{PGK}_Mpro(S2)-15gs-TetR- $_{Mpro}CS_{NAT1}$ -NLS-VP64-pA<br><br>Mammalian expression plasmid encoding SARS-CoV-2 Mpro linked to N-terminal of DNA binding domain TetR fused to transcription activation domain VP64 with fusion linker containing $Mpro_{NAT1}$ cleavage site and NLS. VP64 was amplified from pAB150 using oNF327 and oNF328 to introduce NLS, PCR fragment was digested with BamHI/XbaI and ligated into pNF153 (BamHI/XbaI).	This work
pNF193	P_{PGK}_Mpro(M)-15gs-TetR-$_{Mpro}CS_{OPT}$-NLS-VP64-pA<br><br>Mammalian expression plasmid encoding MERS Mpro linked to N-terminal of DNA binding domain TetR fused to transcription activation domain VP64 with fusion linker containing $Mpro_{OPT}$ cleavage site and NLS. Mpro was amplified from synthetic gene fragment DNA_twist_Mpro(M) using oNF342 and oNF343. TetR was amplified from pTS1106 using oNF260 and oNF251. PCR assembly reaction using oNF342 and oNF251 was used to fuse Mpro(M) and TetR-$_{Mpro}CS_{OPT}$ with 15gs linker, PCR fragment was digested with EcoRI/BamHI and ligated into pNF167 (EcoRI/BamHI).	This work
pNF194	P_{PGK}_Mpro(M)-15gs-TetR-NLS-VP64-pA	This work

	Mammalian expression plasmid encoding MERS Mpro linked to N-terminal of DNA binding domain TetR fused to transcription activation domain VP64 with fusion linker containing NLS. Mpro(M) was amplified from synthetic gene fragment DNA_twist_Mpro(M) using oNF342 and oNF343. TetR was amplified from pTS1106 using oNF260 and oNF261. PCR assembly reaction using oNF342 and oNF261 was used to fuse Mpro(M) and TetR with 15gs linker, PCR fragment was digested with EcoRI/BamHI and ligated into pNF167 (EcoRI/BamHI).	
pNF196	$_{Otet}P_{hCMVmin}$_YPet_sTRSV-pA<br><br>Mammalian reporter plasmid encoding TetR driven expression of YPet with a sTRSV ribozyme-dependent destabilization module in the 3'-UTR. Ypet was excised from bb3-YPet using EcoRI/XbaI and ligated into pNF151 (EcoRI/XbaI).	This work
pNF199	P_{PGK}_Mpro(S2)$_{C145A}$-15gs-TetR-$_{Mpro}CS_{OPT}$-VP64-pA<br><br>Mammalian expression plasmid encoding mutated SARS-CoV-2 $Mpro_{C145A}$ linked to N-terminal of DNA binding domain TetR fused to transcription activation domain VP64 with fusion linker containing $Mpro_{OPT}$ cleavage site and NLS. C145A mutation was introduced by amplifying Mpro(S2) from synthetic gene fragment DNA_twist_Mpro(S2) using oNF258 and oNF349 to amplify N-terminal and oNF348 and oNF259 to amplify C-terminal followed by PCR assembly using oNF258 and oNF259. TetR was amplified from pTS1106 using oNF260 and oNF251 to introduce $Mpro_{OPT}$ cleavage site. PCR assembly reaction using oNF258 and oNF251 was used to fuse Mpro(S2)$_{C145A}$ and TetR-$_{Mpro}CS_{OPT}$ with 15gs linker, PCR fragment was digested with EcoRI/BamHI ligated into pNF167 (EcoRI/ BamHI).	This work
pNF221	P_{PGK}_Mpro(S2)-TetR-$_{Mpro}CS_{NAT2}$-VP64-pA<br><br>Mammalian expression plasmid encoding SARS-CoV-2 Mpro linked to N-terminal of DNA binding domain TetR fused to transcription activation domain VP64 with fusion linker containing $Mpro_{NAT2}$ cleavage site and NLS. Mpro(S2)-15gs-TetR-$_{Mpro}CS_{NAT2}$ was amplified from pNF168 using oNF258 and oNF392 to introduce $MproCS_{NAT2}$, PCR fragment was digested with EcoRI/BamHI and ligated into pNF167 (EcoRI/BamHI).	This work
pNF222	P_{PGK}_Mpro(S2)-TetR-$_{Mpro}CS_{NAT3}$-VP64-pA<br><br>Mammalian expression plasmid encoding SARS-CoV-2 Mpro linked to N-terminal of DNA binding domain TetR fused to transcription activation domain VP64 with fusion linker containing $Mpro_{NAT3}$ cleavage site and NLS. Mpro(S2)-15gs-TetR-$_{Mpro}CS_{NAT3}$ was amplified from pNF168 using oNF258 and oNF393 to introduce $_{Mpro}CS_{NAT3}$, PCR fragment was digested with EcoRI/BamHI and ligated into pNF167 (EcoRI/BamHI).	This work
pNF223	P_{PGK}_Mpro(S2)-TetR-$_{Mpro}CS_{NAT4}$-VP64-pA<br><br>Mammalian expression plasmid encoding SARS-CoV-2 Mpro linked to N-terminal of DNA binding domain TetR fused to transcription activation domain VP64 with fusion linker containing $Mpro_{NAT4}$ cleavage site and NLS. Mpro(S2)-15gs-TetR-$_{Mpro}CS_{NAT4}$ was amplified from pNF168 using oNF258 and oNF394 to introduce $_{Mpro}CS_{NAT4}$, PCR fragment was digested with EcoRI/BamHI and ligated into pNF167 (EcoRI/BamHI).	This work
pNF224	P_{PGK}_Mpro(S2)-TetR-$_{Mpro}CS_{NAT5}$-VP64-pA<br><br>Mammalian expression plasmid encoding SARS-CoV-2 Mpro linked to N-terminal of DNA binding domain TetR fused to transcription activation domain VP64 with fusion linker containing $Mpro_{NAT5}$ cleavage site and NLS. Mpro(S2)-15gs-TetR-$_{Mpro}CS_{NAT5}$ was amplified from	This work

	pNF168 using oNF258 and oNF395 to introduce ${}_{Mpro}CS_{NAT5}$, PCR fragment was digested with EcoRI/BamHI and ligated into pNF167 (EcoRI/BamHI).	
pNF225	P_{PGK}_Mpro(S2)-TetR-${}_{Mpro}CS_{NAT6}$-VP64-pA<br><br>Mammalian expression plasmid encoding SARS-CoV-2 Mpro linked to N-terminal of DNA binding domain TetR fused to transcription activation domain VP64 with fusion linker containing $Mpro_{NAT6}$ cleavage site and NLS. Mpro(S2)-15gs-TetR-${}_{Mpro}CS_{NAT6}$ was amplified from pNF168 using oNF258 and oNF396 to introduce ${}_{Mpro}CS_{NAT6}$, PCR fragment was digested with EcoRI/BamHI and ligated into pNF167 (EcoRI/BamHI).	This work
pNF226	P_{PGK}_Mpro(S2)-TetR-${}_{Mpro}CS_{NAT7}$-VP64-pA<br><br>Mammalian expression plasmid encoding SARS-CoV-2 Mpro linked to N-terminal of DNA binding domain TetR fused to transcription activation domain VP64 with fusion linker containing $Mpro_{NAT7}$ cleavage site and NLS. Mpro(S2)-15gs-TetR-${}_{Mpro}CS_{NAT7}$ was amplified from pNF168 using oNF258 and oNF397 to introduce ${}_{Mpro}CS_{NAT7}$, PCR fragment was digested with EcoRI/BamHI and ligated into pNF167 (EcoRI/BamHI).	This work
pNF227	P_{PGK}_Mpro(S2)-TetR-${}_{Mpro}CS_{NAT8}$-VP64-pA<br><br>Mammalian expression plasmid encoding SARS-CoV-2 Mpro linked to N-terminal of DNA binding domain TetR fused to transcription activation domain VP64 with fusion linker containing $Mpro_{NAT8}$ cleavage site and NLS. Mpro(S2)-15gs-TetR-${}_{Mpro}CS_{NAT8}$ was amplified from pNF168 using oNF258 and oNF398 to introduce ${}_{Mpro}CS_{NAT8}$, PCR fragment was digested with EcoRI/BamHI and ligated into pNF167 (EcoRI/BamHI).	This work
pNF228	P_{PGK}_Mpro(S2)-TetR-${}_{Mpro}CS_{NAT9}$-VP64-pA<br><br>Mammalian expression plasmid encoding SARS-CoV-2 Mpro linked to N-terminal of DNA binding domain TetR fused to transcription activation domain VP64 with fusion linker containing $Mpro_{NAT9}$ cleavage site and NLS. Mpro(S2)-15gs-TetR-${}_{Mpro}CS_{NAT9}$ was amplified from pNF168 using oNF258 and oNF399 to introduce ${}_{Mpro}CS_{NAT9}$, PCR fragment was digested with EcoRI/BamHI and ligated into pNF167 (EcoRI/BamHI).	This work
pNF229	P_{PGK}_Mpro(S2)-TetR-${}_{Mpro}CS_{NAT10}$-VP64-pA<br><br>Mammalian expression plasmid encoding SARS-CoV-2 Mpro linked to N-terminal of DNA binding domain TetR fused to transcription activation domain VP64 with fusion linker containing $Mpro_{NAT10}$ cleavage site and NLS. Mpro(S2)-15gs-TetR-${}_{Mpro}CS_{NAT10}$ was amplified from pNF168 using oNF258 and oNF400 to introduce ${}_{Mpro}CS_{NAT10}$, PCR fragment was digested with EcoRI/BamHI and ligated into pNF167 (EcoRI/BamHI).	This work
pNF230	P_{PGK}_Mpro(S2)-TetR-${}_{Mpro}CS_{NAT11}$-VP64-pA<br><br>Mammalian expression plasmid encoding SARS-CoV-2 Mpro linked to N-terminal of DNA binding domain TetR fused to transcription activation domain VP64 with fusion linker containing $Mpro_{NAT11}$ cleavage site and NLS. Mpro(S2)-15gs-TetR-${}_{Mpro}CS_{NAT11}$ was amplified from pNF168 using oNF258 and oNF401 to introduce ${}_{Mpro}CS_{NAT11}$, PCR fragment was digested with EcoRI/BamHI and ligated into pNF167 (EcoRI/BamHI).	This work
pNF231	P_{PGK}_PLpro(S2)-TetR-${}_{PLpro}CS_{NAT2}$-VP16-pA<br><br>Mammalian expression plasmid encoding SARS-CoV-2 PLpro linked to N-terminal of DNA binding domain TetR fused to transcription	This work

	activation domain VP16 with fusion linker containing $PLpro_{NAT3}$ cleavage site and NLS. PLpro(S2)-15gs-TetR-$_{PLpro}CS_{NAT2}$ was amplified from pNF162 using oNF263 and oNF402 to introduce $_{PLpro}CS_{NAT2}$, PCR fragment was digested with EcoRI/BamHI and ligated into pNF138 (EcoRI/BamHI).	
pNF236	P_{PGK}_Mpro(S2)$_{C156F}$-15gs-TetR-$_{Mpro}CS_{OPT}$-VP64-pA<br><br>Mammalian expression plasmid encoding mutated SARS-CoV-2 $Mpro_{C156F}$ linked to N-terminal of DNA binding domain TetR fused to transcription activation domain VP64 with fusion linker containing $Mpro_{OPT}$ cleavage site and NLS. C156F mutation was introduced by amplifying Mpro(S2) from synthetic gene fragment DNA_twist_Mpro(S2) using oNF258 and oNF440 to amplify N-terminal and oNF439 and oNF259 to amplify C-terminal followed by PCR assembly using oNF258 and oNF259. TetR was amplified from pTS1106 using oNF260 and oNF251 to introduce $Mpro_{OPT}$ cleavage site. PCR assembly reaction using oNF258 and oNF251 was used to fuse Mpro(S2)$_{C156F}$ and TetR-$_{Mpro}CS_{OPT}$ with 15gs linker, PCR fragment was digested with EcoRI/BamHI ligated into pNF167 (EcoRI/ BamHI).	This work
pNF237	P_{PGK}_Mpro(S2)$_{G71S}$-15gs-TetR-$_{Mpro}CS_{OPT}$-VP64-pA<br><br>Mammalian expression plasmid encoding mutated SARS-CoV-2 $Mpro_{G71S}$ linked to N-terminal of DNA binding domain TetR fused to transcription activation domain VP64 with fusion linker containing $Mpro_{OPT}$ cleavage site and NLS. G71S mutation was introduced by amplifying Mpro(S2) from synthetic gene fragment DNA_twist_Mpro(S2) using oNF258 and oNF442 to amplify N-terminal and oNF441 and oNF259 to amplify C-terminal followed by PCR assembly using oNF258 and oNF259. TetR was amplified from pTS1106 using oNF260 and oNF251 to introduce $Mpro_{OPT}$ cleavage site. PCR assembly reaction using oNF258 and oNF251 was used to fuse Mpro(S2)$_{G71S}$ and TetR-$_{Mpro}CS_{OPT}$ with 15gs linker, PCR fragment was digested with EcoRI/BamHI ligated into pNF167 (EcoRI/ BamHI).	This work
pNF238	P_{PGK}_Mpro(S2)$_{H41A}$-15gs-TetR-$_{Mpro}CS_{OPT}$-VP64-pA<br><br>Mammalian expression plasmid encoding mutated SARS-CoV-2 $Mpro_{H41A}$ linked to N-terminal of DNA binding domain TetR fused to transcription activation domain VP64 with fusion linker containing $Mpro_{OPT}$ cleavage site and NLS. H41A mutation was introduced by amplifying Mpro(S2) from synthetic gene fragment DNA_twist_Mpro(S2) using oNF258 and oNF444 to amplify N-terminal and oNF443 and oNF259 to amplify C-terminal, followed by PCR assembly using oNF258 and oNF259. TetR was amplified from pTS1106 using oNF260 and oNF251 to introduce $Mpro_{OPT}$ cleavage site. PCR assembly reaction using oNF258 and oNF251 was used to fuse Mpro(S2)$_{H41A}$ and TetR-$_{Mpro}CS_{OPT}$ with 15gs linker, PCR fragment was digested with EcoRI/BamHI ligated into pNF167 (EcoRI/ BamHI).	This work
pNF239	P_{PGK}_Mpro(S2)$_{P184L}$-15gs-TetR-$_{Mpro}CS_{OPT}$-VP64-pA<br><br>Mammalian expression plasmid encoding mutated SARS-CoV-2 $Mpro_{P184L}$ linked to N-terminal of DNA binding domain TetR fused to transcription activation domain VP64 with fusion linker containing $Mpro_{OPT}$ cleavage site and NLS. P184L mutation was introduced by amplifying Mpro(S2) synthetic gene fragment DNA_twist_Mpro(S2) using oNF258 and oNF446 to amplify N-terminal and oNF445 and oNF259 to amplify C-terminal followed by PCR assembly using oNF258 and oNF259. TetR was amplified from pTS1106 using oNF260 and oNF251 to introduce $Mpro_{OPT}$ cleavage site. PCR assembly reaction using oNF258 and oNF251 was used to fuse Mpro(S2)$_{P184L}$ and TetR-$_{Mpro}CS_{OPT}$ with 15gs linker, PCR fragment was digested with EcoRI/BamHI ligated into pNF167 (EcoRI/ BamHI).	This work

pNF240	P_{PGK}_Mpro(S2)$_{R279C}$-15gs-TetR-$_{Mpro}$CS$_{OPT}$-VP64-pA<br><br>Mammalian expression plasmid encoding mutated SARS-CoV-2 Mpro$_{R279C}$ linked to N-terminal of DNA binding domain TetR fused to transcription activation domain VP64 with fusion linker containing Mpro$_{OPT}$ cleavage site and NLS. R279C mutation was introduced by amplifying Mpro(S2) from synthetic gene fragment DNA_twist_Mpro(S2) using oNF258 and oNF448 to amplify N-terminal and oNF447 and oNF259 to amplify C-terminal followed by PCR assembly using oNF258 and oNF259. TetR was amplified from pTS1106 using oNF260 and oNF251 to introduce Mpro$_{OPT}$ cleavage site. PCR assembly reaction using oNF258 and oNF251 was used to fuse Mpro(S2)$_{R279C}$ and TetR-$_{Mpro}$CS$_{OPT}$ with 15gs linker, PCR fragment was digested with EcoRI/BamHI ligated into pNF167 (EcoRI/ BamHI).	This work
pNF241	P_{PGK}_Mpro(S2)$_{V77A/K90R}$-15gs-TetR-$_{Mpro}$CS$_{OPT}$-VP64-pA<br><br>Mammalian expression plasmid encoding mutated SARS-CoV-2 Mpro$_{V77A/K90R}$ linked to N-terminal of DNA binding domain TetR fused to transcription activation domain VP64 with fusion linker containing Mpro$_{OPT}$ cleavage site and NLS. V77A/K90R mutation was introduced by amplifying Mpro(S2) from synthetic gene fragment DNA_twist_Mpro(S2) using oNF258 and oNF450 to amplify N-terminal and oNF449 and oNF259 to amplify C-terminal, followed by PCR assembly using oNF258 and oNF259. TetR was amplified from pTS1106 using oNF260 and oNF251 to introduce Mpro$_{OPT}$ cleavage site. PCR assembly reaction using oNF258 and oNF251 was used to fuse Mpro(S2)$_{V77A/K90R}$ and TetR-$_{Mpro}$CS$_{OPT}$ with 15gs linker, PCR fragment was digested with EcoRI/BamHI ligated into pNF167 (EcoRI/ BamHI).	This work
pNF242	P_{PGK}_Mpro(S2)$_{W31A}$-15gs-TetR-$_{Mpro}$CS$_{OPT}$-VP64-pA<br><br>Mammalian expression plasmid encoding mutated SARS-CoV-2 Mpro$_{W31A}$ linked to N-terminal of DNA binding domain TetR fused to transcription activation domain VP64 with fusion linker containing Mpro$_{OPT}$ cleavage site and NLS. W31A mutation was introduced by amplifying Mpro(S2) from synthetic gene fragment DNA_twist_Mpro(S2) using oNF451 and oNF259. TetR was amplified from pTS1106 using oNF260 and oNF251 to introduce Mpro$_{OPT}$ cleavage site. PCR assembly reaction using oNF451 and oNF251 was used to fuse Mpro(S2)$_{W31A}$ and TetR-$_{Mpro}$CS$_{OPT}$ with 15gs linker, PCR fragment was digested with EcoRI/BamHI ligated into pNF167 (EcoRI/ BamHI).	This work
pNF245	P_{PGK}_Mpro(S2)-15gs-rTetR-$_{Mpro}$CS$_{OPT}$-VP64-pA<br><br>Mammalian expression plasmid encoding SARS-CoV-2 Mpro linked to N-terminal of DNA binding domain rTetR fused to transcription activation domain VP64 with fusion linker containing Mpro$_{OPT}$ cleavage site and NLS. Mpro(S2) was amplified from synthetic gene fragment DNA_twist_Mpro(S2) using oNF258 and oNF259. rTetR was amplified from BB3-rTetR using oNF455 and oNF456. PCR assembly reaction using oNF258 and oNF456 was used to fuse Mpro(S2) and rTetR-$_{Mpro}$CS$_{OPT}$ with 15gs linker, PCR fragment was digested with EcoRI/BamHI ligated into pNF167 (EcoRI/ BamHI).	This work
pNF246	P_{PGK}_PLpro(S2)-15gs-rTetR-$_{PLpro}$CS$_{NAT2}$-VP16-pA<br><br>Mammalian expression plasmid encoding SARS-CoV-2 PLpro linked to N-terminal of DNA binding domain rTetR fused to transcription activation domain VP16 with fusion linker containing PLpro$_{NAT2}$ cleavage site and NLS. PLpro(S2) was amplified from synthetic gene fragment DNA_twist_Mpro(S2) using oNF263 and oNF272. rTetR was amplified from BB3-rTetR using oNF455 and oNF457. PCR assembly reaction using oNF263 and oNF457 was used to fuse PLpro(S2) and	This work

	rTetR-$_{PLpro}CS_{NAT2}$ with 15gs linker, PCR fragment was digested with EcoRI/BamHI ligated into pNF138 (EcoRI/ BamHI).	
pAna225	P_{5xUAS}_ss-nanoLuc_sTRSV-pA<br><br>Mammalian reporter plasmid encoding Gal4 driven expression of nanoLuc with a sTRSV ribozyme-dependent destabilization module in the 3'-UTR. P_{5xUAS} was excised from BB3-5xUAS-SEAP using BglII/EcoRI and ligated into pNF151 (BglII/EcoRI).	This work
pNF250	P_{PGK}_HCVp-TetR-$_{HCVp}CS$-VP64-pA<br><br>Mammalian expression plasmid encoding hepatitis C virus protease linked to N-terminal of DNA binding domain TetR fused to transcription activation domain VP64 with fusion linker containing HCVp cleavage site and NLS. HCVp was amplified from Addgene plasmid #112628 using oNF468 and oNF469. TetR was amplified from pTS1106 using oNF260 and oNF471. PCR assembly reaction using oNF468 and oNF471 was used to fuse HCVp and TetR-$_{HCVp}CS$ with 15gs linker. The PCR fragment was digested with EcoRI/BamHI and ligated into pNF167 (EcoRI/BamHI).	This work
pNF256	P_{CMV}_FlipGFP-PLproCS-pA<br><br>Mammalian expression plasmid encoding FlipGFP containing PLpro cleavage site. The PLpro cleavage site was introduced by PCR-amplifying Addgene plasmid #124429 using oNF475 and oNF476 and back-ligating the fragment.	This work

Abbreviations

Citrine, improved version of YFP derived from *Aequorea victoria*
15gs, flexible linker containing 15x glycine and serine
YPet, improved version of Venus derived from *Aequorea victoria*
sTRSV, engineered hammerhead ribozyme derived from the natural ribozyme from the satellite RNA of the tobacco ringspot virus
TetR, Tet repressor protein derived from *Escherichia coli* transposon Tn10
rTetR, reverse TetR repressor
SEAP, human, placental secreted alkaline phosphatase
ss-nanoLuc, secreted version of nanoluc luciferase derived from deep sea shrimp *Oplophorus gracilirostris*
P_{TREBI}, bidirectional 3G tetracycline-responsive promoter
O_{TetR}, TetR operator consisting of 7 repeats of TetR binding sites
P_{5xUAS}, GAL4BD operator consisting of 5 repeats of GAL4BD binding sites *Saccharomyces cerevisiae*-derived Gal4-specific upstream activator sequence
P_{hCMV}, human cytomegalovirus immediate early promoter
$P_{hCMVmin}$, minimal human cytomegalovirus immediate early promoter
P_{mPGK}, mouse phosphoglycerate promoter
VPR, VP64-p65-Rta (a fusion of 3 transactivation domains)
VP64, 4 core repeats of VP16 transactivation domain
NLS, nuclear localization sequence
MCS, multiple cloning site
$_{Mpro}CS_{OPT}$, optimized cleavage site for main protease
$_{PLpro}CS_{NAT1-3}$, native cleavage sites recognized by papain-like protease derived from SARS-CoV-2 polyprotein
Mpro(S2), Main protease derived from SARS-CoV-2
PLpro(S2), Papain-like protease derived from SARS-CoV-2

$_{Mpro}$CS$_{NAT1-11}$, native cleavage sites recognized by main protease derived from SARS-CoV-2 polyprotein
Mpro(S1), Main protease derived from SARS-CoV-1
PLpro(S1), Papain-like protease derived from SARS-CoV-1
GAL4BD, GAL4 DNA binding domain
Mpro(M), Main protease derived from MERS
pA, polyadenylation signal
Env140ac, active ribozyme from environmental samples
3'UTR, three prime untranslated region
VP16, herpes simplex virus-derived transactivation domain
Gal4$_{DBD}$, Gal4 transcription factor DNA binding domain

Supplementary Table 5. Oligonucleotides used in this study

Oligonucleotide name	Sequence
oNF11	AGGAAGGATCCGGCCCAAAGAAGAAACGGAAGGTGGGAAGTGGTGCTCCTCCTACAGATGTCAGCCTGGGGG
oNF248	GGCCCTCTAGATTACCCACCGTACTCGTCAATTC
oNF249	TGCTGGAATTCGCCACCATGTCCAGATTAGATAAAAGTAAAGTGATTAAC
oNF250	GGGCCGGATCCCATTTTCCTAAATCCGCTCTGGAGGACGGCGCTTGTACTTCCGGACCCACTTTCACATTTAAG
oNF251	GGGCCGGATCCCATCTTTCTAAACCCGCTTTGGAGCCGGACTGTGGTACTTCCGGACCCACTTTCACATTTAAG
oNF253	GGGCCGGATCCCCAATTATTGACAATCTTGCCGCCTTTCAGAGCGATACTTCCGGACCCACTTTCACATTTAAG
oNF258	TGCTGGAATTCGCCACCATGAGCGGCTTCAGAAAAATGGC
oNF259	ACCGCCTCCGGAGCCTCCGCCCCCGGAGCCTCCGCCTCCGGAACCCTGGAAGGTGACGCCAG
oNF260	GGCTCCGGGGGCGGAGGCTCCGGAGGCGGTTCCAGATTAGATAAAAGTAAAGTGATTAACAGC
oNF261	GGGCCGGATCCACTTCCGGACCCACTTTCACATTTAAGTTGTTTTTC
oNF262	GGCCCTCTAGATTACTGGAAGGTGACGCCAGAG
oNF263	CGTGAGAATTCGCCACCATGTCCTTGCGCGAAGTCCGG
oNF272	ACCGCCTCCGGAGCCTCCGCCCCCGGAGCCTCCGCCTCCGGAACCTCCATCTAGCTTGTACGTGAC
oNF303	GGATCCGGCCCAAAGAAGAAACGGAAGGTGGGAAGTGGTGAGGCCAGCGGTTCCGGACGGGCTGAC
oNF304	ACCACTTCCCACCTTCCGTTTCTTCTTTGGGCCGGATCC
oNF305	GCATGCTCGAGTTAAAACAGAGATGTGTCGAAGATGGACAG
oNF312	GGTTCCGGAGGCGGAGGCTCCGGGGGCGGAGGCTCCGGAGGCGGTATGAAGCTACTGTCTTCTATCGAAC
oNF326	GGGCCGGATCCACTTCCCGATACAGTCAACTGTCTTTGAC
oNF327	AGATGGGATCCGGCCCAAAGAAGAAACGGAAGGTGGGAAGTGGTGAGGCCAGCGGTTCCGGACGGGCTGAC
oNF328	GGCCCTCTAGATGCATGTTACCTAGAGTTAATCAGCATGTCCAG
oNF332	AGACCGAATTCGCCACCATGTCAGGCTTTAGGAAGATGG
oNF333	ACCGCCTCCGGAGCCTCCGCCCCCGGAGCCTCCGCCTCCGGAACCCTGAAATGTCACACCGGAGC
oNF334	GACCGAATTCGCCACCATGGAGGTAAAGACAATTAAAGTCTTTAC
oNF335	ACCGCCTCCGGAGCCTCCGCCCCCGGAGCCTCCGCCTCCGGAACCTTTGATAGTGGTAGTATAACTTGTTTC
oNF340	GGGCCGGATCCCATCTTTCTAAACCCGCTTTGGAGCCGGACTGTGGTACTTCCCGATACAGTCAACTGTCTTTGACC

oNF342	GAGACCGAATTCGCCACCATGTCGGGCTTGGTGAAAATG
oNF343	ACCGCCTCCGGAGCCTCCGCCCCCGGAGCCTCCGCCTCCGGAACCCTGCATGACC ACGCCCATAATC
oNF348	CTCATTTCTGAATGGCAGCGCAGGTAGTGTGGGGTTTAAC
oNF349	GTTAAACCCCACACTACCTGCGCTGCCATTCAGAAATGAG
oNF392	GGGCCGGATCCTGTCCTTTCACTGCGGATTGAAATGTCACGCCTGAACTTCCGG ACCCACTTTCAC
oNF393	GGGCCGGATCCAACATCTGACATCTTTGACTGCACGGTCGCGACCTTACTTCCGG ACCCACTTTCAC
oNF394	GGGCCGGATCCGAACTCAGAAGCGATAGCTTGCAATGTTGCGCGATTACTTCCGG ACCCACTTTCAC
oNF395	GGGCCGGATCCGGGAGACAGTTCATTGTTTTGGAGCTTAACTGCGCTACTTCCGG ACCCACTTTCAC
oNF396	GGGCCGGATCCTTCCGTGGCATTGCCTGCCTGAAGCCTGACGGTAGCACTTCCGG ACCCACTTTCAC
oNF397	GGGCCGGATCCAGACTGTGCATCCGCAGACTGCAACATGGGCTCTCTACTTCCGG ACCCACTTTCAC
oNF398	GGGCCGGATCCTACGCAGGCACCCACCGCTTGCAGCACAGTGTGTGGACTTCCGG ACCCACTTTCAC
oNF399	GGGCCGGATCCCCCAGTTACGTTTTCAGCCTGGAGTGTAGCGACGTTACTTCCGG ACCCACTTTCAC
oNF400	GGGCCGGATCCCGCGACATTCTCCAAGGACTGCAGCCTGGTAAACGTACTTCCGG ACCCACTTTCAC
oNF401	GGGCCGGATCCTTGCCAAGCCTGAGAGCTTTGCAACTTGGGATAGAAACTTCCGG ACCCACTTTCAC
oNF402	GGGCCGGATCCAGTTACTTTGGTAGGAGCGCCCCCCTTCAACGTGAAACTTCCGG ACCCACTTTCAC
oNF439	GGGGTTTAACATTGATTACGACTTTGTTTCTTTCTGTTATATGC
oNF440	GCATATAACAGAAAGAAACAAAGTCGTAATCAATGTTAAACCCC
oNF441	CTTAGTTCAGGCTAGCAATGTGCAGTTGCG
oNF442	CGCAACTGCACATTGCTAGCCTGAACTAAG
oNF443	GCCCCCGTGCTGTGATTTGTACCAGCGAGG
oNF444	CCTCGCTGGTACAAATCACAGCACGGGGGC
oNF445	GGAACTTTTACGGCCTCTTTGTGGACCGGC
oNF446	GCCGGTCCACAAAGAGGCCGTAAAAGTTCC
oNF447	GAATTGCTGCAGAACGGAATGAACGGATGCACTATTC
oNF448	GAATAGTGCATCCGTTCATTCCGTTCTGCAGCAATTC
oNF449	CAGTTGCGGGCTATTGGCCACAGCATGCAAAACTGCGTTCTAAAACTCAGGGTAG ACACC
oNF450	GGTGTCTACCCTGAGTTTTAGAACGCAGTTTTGCATGCTGTGGCCAATAGCCCGC AACTG
oNF451	CCGAATTCGCCACCATGAGCGGCTTCAGAAAAATGGCTTTTCCCTCGGGCAAAGT GGAGGGGTGCATGGTGCAGGTGACCTGCGGAACTACTACATTGAACGGCCTTGCT CTCGATGATG
oNF455	GGTTCCGGAGGCGGAGGCTCCGGGGGCGGAGGCTCCGGAGGCGGTTCAAGACTG GACAAGAGCAAAG
oNF456	GGGCCGGATCCCATCTTTCTAAACCCGCTTTGGAGCCGGACTGTGGTACTTCCGG AGCCGCTTTCGCACTTTAG
oNF457	GGGCCGGATCCCCAATTATTGACAATCTTGCCGCCTTTCAGAGCGATACTTCCGG AGCCGCTTTCGCACTTTAG
oNF468	GACCGAATTCGCCACCATGTCCGGGACAGGCTGCGTGGTCATAGTGGGCAGGATC GTCTTGTCCGGCTCCGGCACTTCCGCGCCCATC
oNF469	ACCGCCTCCGGAGCCTCCGCCCCCGGAGCCTCCGCCTCCGGAACCGAACTCCTGG TAGAGAACCTCCC
oNF471	GGGCCGGATCCGTGCTGAGAGCACTCTTCCATCTCATCGAACTCACTTCCGGACC CACTTTCAC
oNF475	GCCCCCCCTCAATGATGCATCGGTAATGCCAGC
oNF476	GCTCCTACCAAAAAGGTGTCCGCCCTGAAG

Supplementary Table 6. Synthetic DNA fragments used in this study.

Fragment name	Sequence
DNA_twist_<br>Mpro(S2)	GCGGAATTCACCATGACTAGTAGCGGCTTCAGAAAAATGGCTTTTCCCTCGGGCA<br>AAGTGGAGGGGTGCATGGTGCAGGTGACCTGCGGAACTACTACATTGAACGGCC<br>TTTGGCTCGATGATGTAGTCTACTGCCCCCGTCACGTGATTTGTACCAGCGAGGAT<br>ATGCTCAATCCTAATTACGAGGACCTGCTTATCAGGAAGTCAAACCATAATTTCTT<br>AGTTCAGGCTGGTAATGTGCAGTTGCGGGTGATTGGCCACAGCATGCAAAACTGC<br>GTTCTAAAACTCAAGGTAGACACCGCCAATCCCAAAACTCCAAAGTATAAGTTTG<br>TCAGAATCCAGCCAGGGCAGACGTTCTCCGTCCTTGCCTGTTACAACGGCTCCCC<br>CTCCGGAGTGTATCAATGTGCAATGCGCCCAAATTTCACCATAAAGGGCTCATTT<br>CTGAATGGCAGCTGTGGTAGTGTGGGGTTTAACATTGATTACGACTGTGTTTCTTT<br>CTGTTATATGCATCACATGGAACTCCCTACAGGTGTTCATGCCGGGACAGACCTG<br>GAGGGGAACTTTTACGGCCCTTTTGTGGACCGGCAGACAGCGCAAGCCGCAGGA<br>ACAGACACAACTATCACCGTTAATGTGCTGGCCTGGCTGTATGCAGCCGTCATCA<br>ACGGGGATCGCTGGTTCTTGAACCGATTTACCACGACACTAAATGATTTCAACCT<br>GGTGGCAATGAAGTACAATTATGAGCCTTTAACGCAGGACCACGTCGATATCCTC<br>GGACCGCTGTCCGCTCAGACTGGTATCGCCGTCCTTGATATGTGCGCTTCTCTCAA<br>AGAATTGCTGCAGAACGGAATGAACGGAAGGACTATTCTGGGAAGTGCGCTGCT<br>GGAAGACGAATTTACCCCATTCGACGTAGTGAGGCAATGCTCTGGCGTCACCTTC<br>CAGgctagcTAAG
DNA_twist_<br>PLpro(S2)	GCGGAATTCACCATGACTAGTTCCTTGCGCGAAGTCCGGACGATTAAGGTGTTTA<br>CAACTGTTGATAATATAAATTTACACACCCAGGTCGTGGACATGAGCATGACATA<br>CGGACAGCAATTCGGGCCCACTTATCTGGATGGGGCCGACGTGACCAAGATAAA<br>ACCCCACAATTCACACGAAGGCAAAACCTTTTACGTGTTGCCAAACGACGATACA<br>CTCAGGGTGGAAGCCTTTGAGTACTACCATACCACCGACCCATCCTTCCTTGGTCG<br>ATATATGTCGGCTCTTAATCATACTAAGAAGTGGAAATACCCCCAGGTGAACGGC<br>TTGACTTCTATCAAGTGGGCTGACAATAACTGCTATCTCGCGACTGCACTCCTCAC<br>CCTGCAGCAGATTGAGCTGAAATTCAATCCTCCGGCGCTGCAGGACGCATACTAT<br>CGTGCCAGGGCCGGAGAAGCTGCAAACTTCTGCGCCCTGATCCTGGCTTATTGTA<br>ACAAAACTGTGGGTGAGCTGGGCGACGTGAGAGAGACTATGTCATACTTGTTCCA<br>GCACGCTAATCTGGATTCCTGTAAAAGAGTGTTAAACGTAGTTTGCAAGACCTGT<br>GGGCAGCAGCAAACAACTCTGAAAGGGGTAGAGGCAGTCATGTATATGGGCACC<br>CTGAGTTATGAACAGTTCAAGAAAGGGGTTCAGATTCCTTGCACCTGCGGCAAAC<br>AGGCCACAAAGTATCTGGTCCAACAGGAGTCCCCCTTTGTTATGATGAGTGCCCC<br>TCCTGCTCAATACGAGCTAAAGCACGGAACATTTACGTGTGCCAGCGAGTACACA<br>GGCAACTACCAATGTGGTCATTACAAACATATCACCAGCAAGGAAACACTTTATT<br>GCATTGATGGAGCACTTCTCACGAAGTCTAGCGAATATAAAGGCCCAATCACAGA<br>CGTGTTTTATAAGGAGAACTCTTACACCACCACAATCAAGCCAGTCACGTACAAG<br>CTAGATGGAgctagcTAAG
DNA_twist_<br>Mpro(S1)	TAAGGAATTCACCATGTCAGGCTTTAGGAAGATGGCCTTTCCTTCCGGCAAAGTG<br>GAGGGATGCATGGTACAGGTCACCTGTGGCACTACTACATTGAATGGCCTATGGC<br>TCGATGACACAGTGTACTGCCCGAGGCACGTGATTTGTACAGCGGAGGACATGCT<br>CAATCCAAACTACGAAGATCTTTTGATAAGAAAGAGCAATCATAGCTTTCTGGTC<br>CAGGCAGGAAACGTGCAGCTGAGAGTGATCGGACATTCGATGCAGAACTGTCTG<br>TTGAGATTGAAAGTTGATACTAGCAACCCCAAGACCCCCAAATATAAATTCGTCC<br>GGATTCAACCTGGCCAAACATTCAGTGTGCTCGCGTGCTACAATGGATCTCCATC<br>TGGTGTTTACCAGTGTGCAATGCGACCTAACCACACCATCAAGGGGTCCTTCCTC<br>AATGGCTCCTGCGGTTCAGTTGGGTTCAACATCGACTACGATTGTGTGAGTTTCTG<br>CTACATGCACCATATGGAATTACCCACCGGTGTGCACGCTGGCACTGATCTGGAA<br>GGGAAGTTCTATGGCCCTTTCGTTGACCGCCAGACAGCACAAGCCGCCGGAACTG<br>ATACCACCATAACGCTTAACGTACTGGCCTGGCTGTATGCAGCTGTCATCAATGG<br>AGATCGGTGGTTCCTGAATAGGTTTACTACTACCCTAAACGACTTCAACCTTGTCG<br>CTATGAAATATAACTATGAGCCCCTCACCCAGGACCACGTCGACATTCTGGGGCC<br>ACTGAGCGCCCAAACAGGGATCGCCGTGTTAGACATGTGTGCCGCTCTGAAGGAA<br>CTGCTTCAGAATGGAATGAATGGCCGCACGATCCTCGGGTCTACGATTCTGGAGG<br>ACGAGTTTACCCCATTTGATGTGGTACGTCAGTGCTCCGGTGTGACATTTCAGgcta<br>gcgGATCCACCGGTgTCTAGATAAG

DNA_twist_ PLpro(S1)	TAAGGAATTCACCATGGAGGTAAAGACAATTAAAGTCTTTACTACTGTAGACAATACCAATCTCCATACGCAGCTGGTCGACATGTCCATGACATATGGGCAGCAATTCGGACCTACATATCTGGACGGTGCCGATGTAACCAAAATTAAGCCCCACGTCAATCATGAGGGCAAAACATTCTTCGTCCTGCCCTCTGACGATACCCTACGATCCGAGGCTTTTGAATATTATCATACCCTGGACGAATCATTTCTGGGGCGGTATATGAGCGCCCTGAACCATACCAAGAAATGGAAGTTCCCACAGGTTGGCGGCCTCACTAGCATCAAGTGGGCTGATAACAACTGCTACTTGTCCTCGGTCTTGCTTGCTCTCCAGCAGCTGGAGGTGAAATTCAATGCCCCCGCGCTCCAGGAGGCATACTACAGAGCAAGGGCCGGAGATGCCGCGAACTTCTGCGCACTGATCCTTGCGTACTCTAATAAGACAGTTGGAGAATTGGGCGACGTGAGGGAAACCATGACGCACCTACTGCAGCACGCCAACTTAGAGAGCGCAAAGCGCGTGCTGAACGTGGTTTGCAAGCACTGTGGACAGAAAACCACTACTCTTACGGGTGTGGAGGCTGTGATGTACATGGGGACTTTGAGCTACGATAACCTGAAAACCGGCGTGTCAATTCCTTGTGTTTGTGGCCGTGACGCTACGCAGTACCTCGTGCAACAGGAATCCAGTTTTGTGATGATGTCTGCCCCACCGGCCGAGTACAAGTTACAGCAAGGCACCTTTCTCTGTGCAAATGAATATACCGGGAATTACCAATGCGGGCACTACACACATATCACAGCTAAGGAGACACTGTACAGAATAGACGGTGCCCACCTTACAAAGATGAGTGAGTATAAGGGACCTGTGACCGATGTGTTTTATAAAGAAACAAGTTATACTACCACTATCAAAgctagcgGATCCACCGGTgTCTAGATAAG
DNA_twist_ Mpro(M)	TAAGGAATTCACCATGTCGGGCTTGGTGAAAATGAGTCACCCAAGCGGGGACGTGGAGGCATGTATGGTGCAGGTGACTTGTGGTTCTATGACTCTTAATGGACTGTGGCTCGACAACACTGTGTGGTGCCCTCGACATGTGATGTGCCCTGCCGATCAACTGAGCGACCCCAACTATGATGCCTTACTCATTTCAATGACCAATCACTCATTTAGCGTCCAGAAGCACATTGGGGCACCAGCCAACCTGAGAGTCGTTGGCCACGCCATGCAGGGCACTCTGCTAAAATTGACAGTGGATGTAGCAAATCCCTCTACCCCCGCCTACACTTTCACCACAGTGAAGCCAGGAGCTGCATTCTCCGTGTTAGCCTGCTATAATGGTCGCCCTACAGGAACCTTCACCGTAGTCATGAGGCCGAATTACACTATAAAGGGTCCTTTCTCTGCGGCAGCTGCGGAAGCGTGGGGTACACAAAGGAGGGATCTGTGATCAACTTCTGTTATATGCACCAAATGGAACTGGCGAATGGCACCCATACCGGCTCTGCATTCGATGGGACCATGTATGGCGCTTTCATGGACAAACAAGTCCATCAGGTGCAGCTGACAGATAAATACTGTAGTGTGAATGTTGTGGCTTGGCTTTACGCCGCGATACTGAATGGCTGTGCTTGGTTCGTTAAGCCCAACCGGACTTCCGTAGTATCATTTAACGAGTGGGCTCTGGCTAACCAGTTTACGGAATTTGTTGGAACACAGTCCGTCGATATGTTGGCGGTTAAGACGGGTGTCGCCATCGAACAGCTCCTCTATGCCATCCAGCAGCTGTACACCGGTTTCCAAGGAAAACAAATCCTTGGGAGTACAATGCTAGAGGACGAATTTACGCCTGAGGACGTTAACATGCAGATTATGGGCGTGGTCATGCAGgctagcgGATCCTAAG

Supplementary Table 7. P-Values, t-values and degrees of freedom for two-sided T-tests

Figure	Compared groups	Fold change	P-Value	Welch-corrected t-values (t) and degrees of freedom (df)
2a	0 µM vs. 1 µM GRL-0617	1.4	0.0002	t=6.653, df=7.412
2a	0 µM vs. 10 µM GRL-0617	7.9	4.41×10^{-8}	t=45.26, df=5.315
2a	0 µM vs. 25 µM GRL-0617	14	4.95×-10^{-7}	t=32.17, df=5.042
2a	0 µM vs. 50 µM GRL-0617	36	8.93×10^{-9}	t=71.96, df=5.031
2a	0 µM vs. 100 µM GRL-0617	40	5.22×10^{-7}	t=32.37, df=5.005
2d	2 h; 0 µM vs. 50 µM GRL-0617	2.1	0.0572	t=3.427, df=2.427
2d	4 h; 0 µM vs. 50 µM GRL-0617	4.3	0.0047	t=13.86, df=2.051
2d	6 h; 0 µM vs. 50 µM GRL-0617	9.7	0.0056	t=13.26, df=2.007

2d	8 h; 0 µM vs. 50 µM GRL-0617	12	0.0024	t=20.17, df=2.008
2d	10 h; 0 µM vs. 50 µM GRL-0617	13	0.0030	t=17.79, df=2.016
2e	hMSC-TERT; 0 µM vs. 100 µM GRL-0617	10	0.0014	t=18.42, df=2.320
2e	HEK293T; 0 µM vs. 100 µM GRL-0617	38	0.0033	t=17.36, df=2.003
2e	HT-1080; 0 µM vs. 100 µM GRL-0617	2.4	0.0289	t=5.293, df=2.153
2e	BHK-21; 0 µM vs. 100 µM GRL-0617	7.0	0.0009	t=16.54, df=2.686
2e	HeLa; 0 µM vs. 100 µM GRL-0617	5.0	0.0107	t=9.586, df=2.002
2e	HepG2; 0 µM vs. 100 µM GRL-0617	6.1	0.0170	t=7.373, df=2.038
2f	0 µM vs. 2 µM GRL-0617	8.2	0.0003	t=55.20, df=2.013
2f	0 µM vs. 10 µM GRL-0617	42	0.0009	t=33.24, df=2.000
2f	0 µM vs. 25 µM GRL-0617	120	0.0029	t=18.50, df=2.000
2f	0 µM vs. 100 µM GRL-0617	106	0.0023	t=20.67, df=2.000
3a	0 µM vs. 1 µM GC-376	1.4	0.0001	t=6.287, df=9.679
3a	0 µM vs. 10 µM GC-376	3.4	2.31×10^{-9}	t=34.42, df=7.317
3a	0 µM vs. 50 µM GC-376	7.2	4.20×10^{-8}	t=42.21, df=5.482
3a	0 µM vs. 100 µM GC-376	13	7.93×10^{-9}	t=63.50, df=5.287
3a	0 µM vs. 200 µM GC-376	19	1.79×10^{-8}	t=60.07, df=5.105
3b	VP16; 0 µM vs. 200 µM GC-376	9.6	0.0058	t=12.89, df=2.015
3b	VP64; 0 µM vs. 200 µM GC-376	15	0.0006	t=39.89, df=2.015
3b	VPR; 0 µM vs. 200 µM GC-376	4.7	0.0014	t=23.34, df=2.117
3c	2 h; 0 µM vs. 100 µM GC-376	1.6	0.0746	t=2.956, df=2.505
3c	4 h; 0 µM vs. 100 µM GC-376	2.5	0.0230	t=6.272, df=2.050
3c	6 h; 0 µM vs. 100 µM GC-376	4.3	0.0008	t=24.47, df=2.284
3c	8 h; 0 µM vs. 100 µM GC-376	5.4	0.0048	t=13.60, df=2.057
3c	10 h; 0 µM vs. 100 µM GC-376	7.2	0.0001	t=41.33, df=2.467
3d	Mpro (SARS-CoV); 0 µM vs. 100 µM GC-376	10	0.0032	t=17.05, df=2.030
3d	Mpro (SARS-CoV); 0 µM vs. 200 µM GC-376	23	0.0024	t=20.08, df=2.007
3d	Mpro (MERS); 0 µM vs. 100 µM GC-376	3.7	0.0028	t=14.28, df=2.276
3d	Mpro (MERS); 0 µM vs. 200 µM GC-376	7.4	7.29×10^{-5}	t=47.85, df=2.572
4b	WT vs. W31A	6.8	0.0104	t=9.598, df=2.019

4b	WT vs. H41A	41	0.0017	t=24.33, df=2.003
4b	WT vs. C145A	41	0.0006	t=40.70, df=2.007
4c	WT vs. C156F - US	1.8	0.0165	t=6.039, df=2.414
4c	WT vs. G71S - CH	1.7	0.0024	t=8.558, df=3.283
4c	WT vs. R279C - FR	2.0	0.0028	t=10.34, df=2.750
4c	WT vs. V77A/K90R - EN	3.9	0.0089	t=9.863, df=2.081
4c	WT vs. P184L - EN	8.5	0.0131	t=8.597, df=2.009
5g	0 µM vs. 2 µM GC-376	1.4	0.0062	t=6.524, df=3.171
5g	0 µM vs. 10 µM GC-376	3.3	0.0006	t=40.89, df=2.001
5g	0 µM vs. 100 µM GC-376	31	3.16×10^{-5}	t=118.0, df=2.201
5g	0 µM vs. 200 µM GC-376	45	0.0005	t=45.60, df=2.013
5h	nanoLuc; (- GC-376, - GRL-0617) vs. (+ GC-376, - GRL-0617)	19	0.0001	t=80.27, df=2.086
5h	SEAP; (- GC-376, - GRL-0617) vs. (+ GC-376, - GRL-0617)	1.0	0.9212	t=0.1055, df=3.856
5h	nanoLuc; (- GC-376, - GRL-0617) vs. (- GC-376, + GRL-0617)	1.0	0.5302	t=0.6910, df=3.732
5h	SEAP; (- GC-376, - GRL-0617) vs. (- GC-376, + GRL-0617)	15	0.0020	t=21.37, df=2.029
5h	nanoLuc; (- GC-376, - GRL-0617) vs. (+ GC-376, + GRL-0617)	14	0.0009	t=32.29, df=2.023
5h	SEAP; (- GC-376, - GRL-0617) vs. (+ GC-376, + GRL-0617)	20	0.0004	t=43.09, df=2.067
6b	Vehicle vs. GRL-0617	2.4	0.0375	t=2.599, df=6.559
6c	Vehicle vs. GC-376	1.9	0.0215	t=5.624, df=2.316
6d	9 h; vehicle vs. 1 mg GRL-0617	1.3	0.0292	t=2.566, df=9.486
6d	9 h; vehicle vs. 2.5 mg GRL-0617	1.8	0.0017	t=4.905, df=7.078
6d	9 h; 1 mg vs. 2.5 mg GRL-0617	1.4	0.0169	t=2.996, df=8.115
6d	12 h; vehicle vs. 1 mg GRL-0617	1.2	0.1431	t=1.596, df=9.493
6d	12 h; vehicle vs. 2.5 mg GRL-0617	1.4	0.0040	t=3.723, df=9.870
6d	24 h; vehicle vs. 1 mg GRL-0617	0.9	0.4134	t=0.8722, df=6.655
6d	24 h; vehicle vs. 2.5 mg GRL-0617	1.0	0.9888	t=0.01464, df=5.792
6e	vehicle vs. GRL-0617	16	0.0322	t=3.186, df=4.101

S2	pLeo665; 0 µM vs. 25 µM GRL-0617	2.3	0.0016	t=20.66, df=2.161
S2	pNF151; 0 µM vs. 100 µM GRL-0617	25	0.0021	t=21.25, df=2.015
S4	0 µM vs. 1 µM GRL-0617	1.1	0.1486	t=1.914, df=3.099
S4	0 µM vs. 10 µM GRL-0617	0.8	0.0625	t=2.922, df=2.959
S4	0 µM vs. 25 µM GRL-0617	0.72	0.0271	t=4.086, df=2.962
S4	0 µM vs. 50 µM GRL-0617	0.62	0.0212	t=6.133, df=2.158
S4	0 µM vs. 100 µM GRL-0617	0.54	0.0147	t=7.631, df=2.093
S8	nanoLuc; (- GC-376, - GRL-0617) vs. (+ GC-376, - GRL-0617)	4.5	0.0001	t=38.44, df=2.524
S8	SEAP; (- GC-376, - GRL-0617) vs. (+ GC-376, - GRL-0617)	1.1	0.7029	t=0.4169, df=3.241
S8	nanoLuc; (- GC-376, - GRL-0617) vs. (- GC-376, + GRL-0617)	1.0	0.7874	t=0.2944, df=3.043
S8	SEAP; (- GC-376, - GRL-0617) vs. (- GC-376, + GRL-0617)	8.5	0.0003	t=29.29, df=2.479
S8	nanoLuc; (- GC-376, - GRL-0617) vs. (+ GC-376, + GRL-0617)	4.4	0.0002	t=35.51, df=2.484
S8	SEAP; (- GC-376, - GRL-0617) vs. (+ GC-376, + GRL-0617)	10.5	0.0017	t=20.56, df=2.136

Supplementary Figures

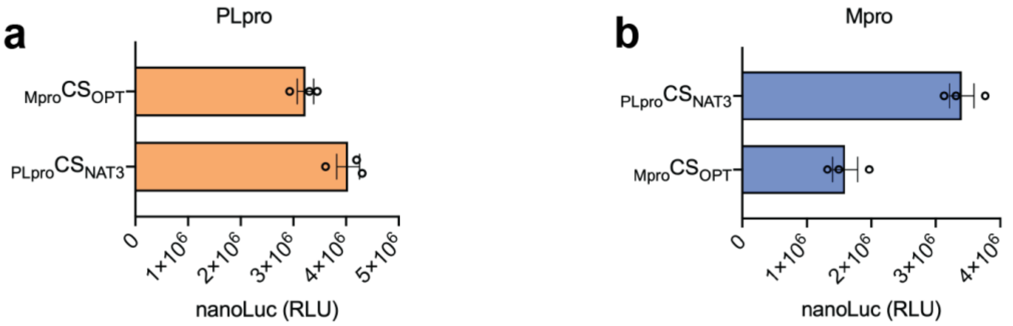

Supplementary Figure 1. *Cleavage efficiency in trans.* **a** Performance of the PLpro gene switch when PLpro is provided in trans in a separate plasmid. NanoLuc expression level from cells co-expressing PLpro and synTF containing either a cognate CS (TetR-$_{PLpro}CS_{NAT3}$-VP16) or a non-cognate CS (TetR-$_{Mpro}CS_{OPT}$-VP16). **b** Performance of the Mpro gene switch when Mpro is provided in trans. NanoLuc expression level from cells co-expressing Mpro and synTF containing either a cognate CS (TetR-$_{Mpro}CS_{OPT}$-VP16) or a non-cognate CS (TetR-$_{PLpro}CS_{NAT3}$-VP16). Data are shown as mean ± SEM, with individual data points, n = 3 biological replicates. Source data for this figure is provided in a Source Data file.

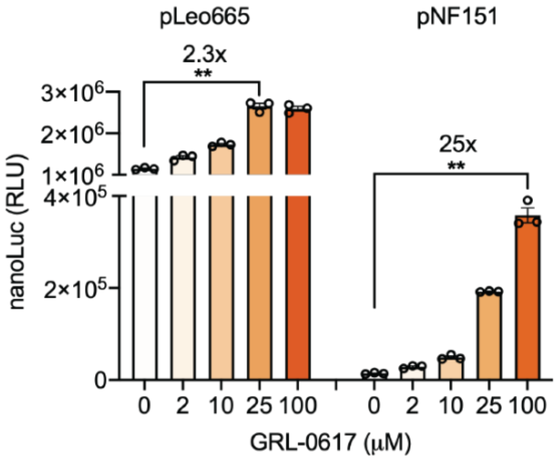

Supplementary Figure 2. *Reporter optimization*. Inducibility comparison between nanoLuc reporter without destabilizing 3'UTR (pLeo665) and reporter containing sTRSV destabilizing hammerhead in the 3'UTR (pNF151). Data are shown as mean ± SEM, with individual data points, n = 3 biological replicates. Statistical significance was calculated by means of Welch's two-tailed t test, **P < 0.01; exact P values are provided in Supplementary Table 7. The numbers above the bars indicate fold difference in reporter expression level, calculated by dividing the mean expression level in the presence of inducer by the mean reporter expression level in the absence of inducer. Source data for this figure is provided in a Source Data file.

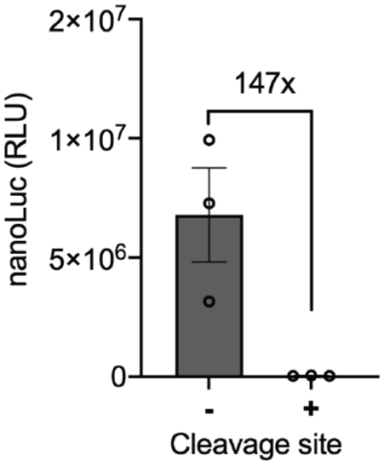

Supplementary Figure 3. *Cleavage efficiency of SARS-CoV derived PLpro***.** Luminescence from secreted nanoLuc when HEK293T cells were transfected with PLpro(S1)-based TAGS bearing CS_{NAT3} from the SARS-CoV-2 polyprotein. A PLpro(S1)-TAGS without CS was used as a control. The number above the bars indicates fold difference in reporter expression level, calculated by dividing the mean expression level in the PLpro(S1)-TAGS without CS by the mean reporter expression level in the PLpro(S1)-TAGS with CS. Data are shown as mean ± SEM, with individual data points, n = 3 biological replicates. Source data for this figure is provided in a Source Data file.

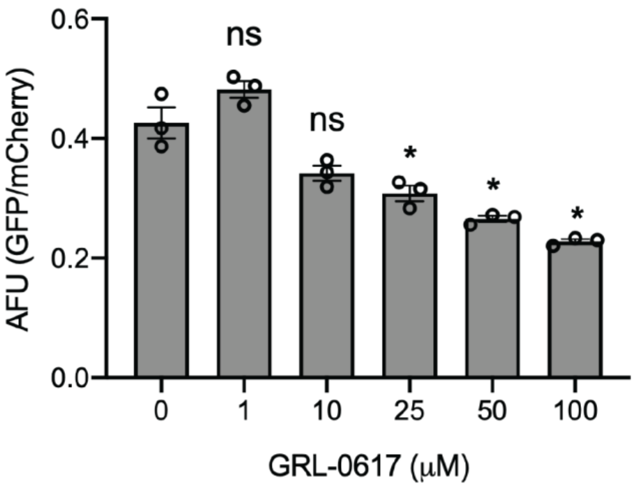

Supplementary Figure 4. *Sensitivity and dose-response of FlipGFP-PLpro to PLpro inhibitor GRL-0617*. Cells transfected with pNF124 and pNF256 were treated with various concentrations of GRL-0617 for 48 h before measuring the fluorescence intensity of GFP and mCherry. AFU was calculated by dividing the GFP signal intensity by the mCherry signal intensity. Data are shown as mean ± SEM, with individual data points, n = 3 biological replicates. Statistical significance was calculated by means of Welch's two-tailed t test, *P < 0.05, ns, not significant; exact P values are provided in Supplementary Table 7. Source data for this figure is provided in a Source Data file.

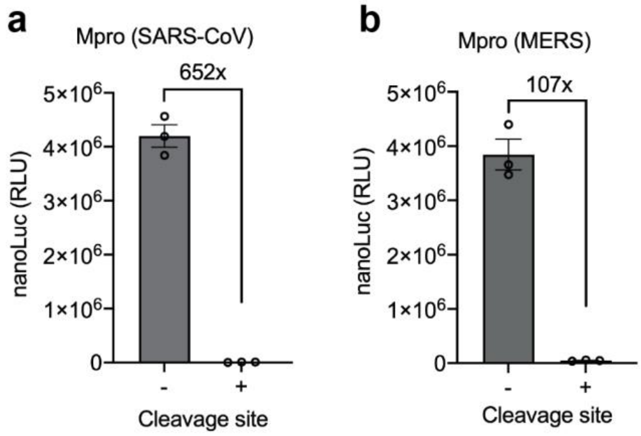

Supplementary Figure 5. *Cleavage efficiency of SARS-CoV and MERS derived Mpro***.** Luminescence from secreted nanoLuc when HEK293T cells were transfected with **a** Mpro(S1) and **b** Mpro(M) based TAGS bearing CS_{OPT} from the SARS-CoV-2 polyprotein. **a** Mpro(S1) and **b** Mpro(M) TAGS without CS were used as controls. The numbers above the bars indicate fold difference in reporter expression level, calculated by dividing the mean expression level in the TAGS without CS by the mean reporter expression level in the TAGS with CS. Data are shown as mean ± SEM, with individual data points, n = 3 biological replicates. Source data for this figure is provided in a Source Data file.

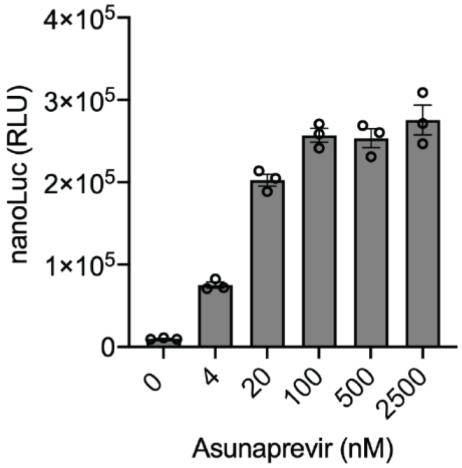

Supplementary Figure 6. *Inducibility and sensitivity of HCVp-TAGS.* HCVp-TAGS-transfected cells (pNF151 and pNF250) were treated with different concentrations of the HCVp inhibitor asunaprevir for 24 h before quantifying nanoLuc expression level. Data are shown as mean ± SEM, with individual data points, n = 3 biological replicates. Source data for this figure is provided in a Source Data file.

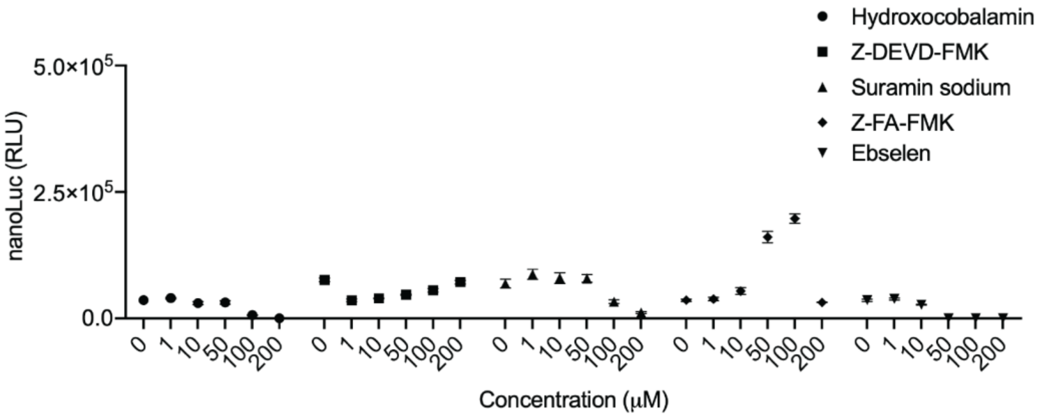

Supplementary Figure 7. *Scoring Mpro activity in the presence of various compounds previously reported to inhibit Mpro*. Mpro-TAGS-transfected HEK293T cells were treated with various concentrations of selected Mpro inhibitors for 24 h before quantifying nanoLuc expression level. Data are shown as mean ± SEM, n = 3 biological replicates. Source data for this figure is provided in a Source Data file.

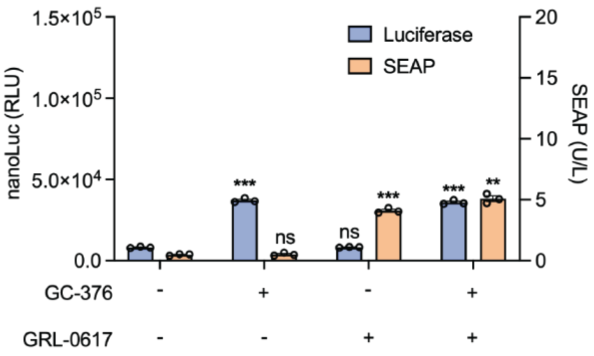

Supplementary Figure 8. *Multiplexing Mpro- and PLpro-based TAGS*. Mpro-TAGS controlling nanoLuc expression and PLpro-TAGS controlling SEAP expression were co-transfected into HEK293T cells, which were then treated with combinations of GC-376 and GRL-0617 at 10 μM for 24 h before profiling reporter expression level. Data are shown as mean ± SEM, with individual data points, n = 3 biological replicates. Statistical significance was calculated by means of Welch's two-tailed t test, **P < 0.01, ***P < 0.001, ns, not significant; exact P values are provided in Supplementary Table 7. Source data for this figure is provided in a Source Data file.

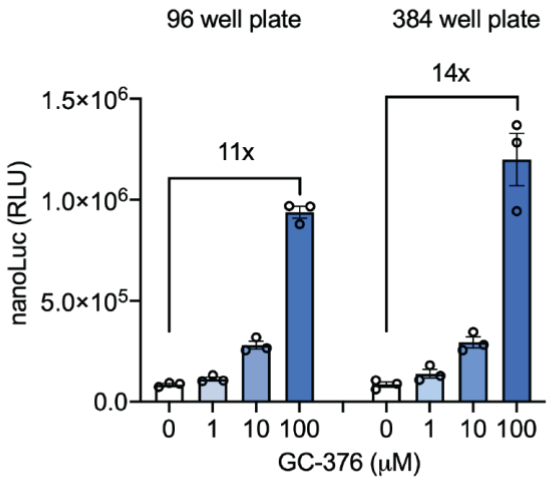

Supplementary Figure 9. *Mpro-TAGS performance in different plate formats*. Mpro-TAGS-transfected HEK293T cells were seeded in either 96-well or 384-well plates before induction with different GC-376 concentrations for 24 h, after which nanoLuc expression level was quantified in the media. Data are shown as mean ± SEM, with individual data points, n = 3 biological replicates. The numbers above the bars indicate fold difference in reporter expression level, calculated by dividing the mean expression level in the presence of inducer by the mean reporter expression level in the absence of inducer. Source data for this figure is provided in a Source Data file.

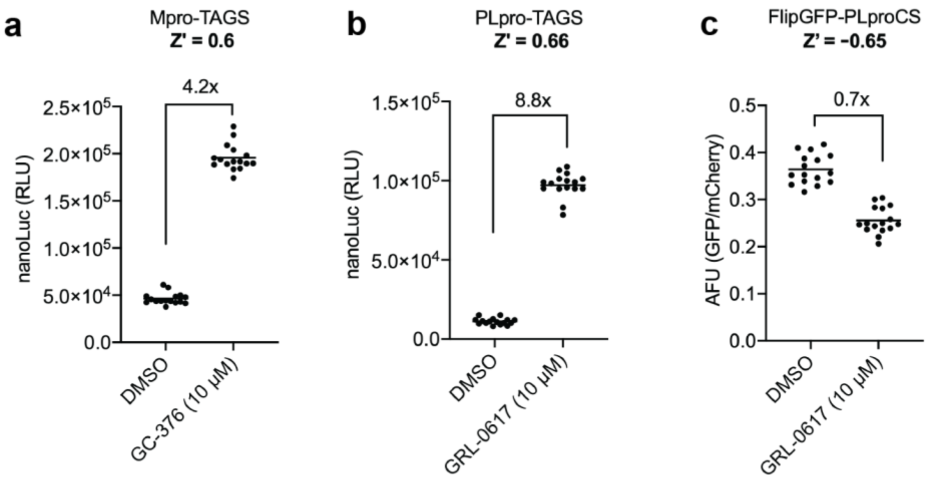

Supplementary Figure 10. *Z' factor values for three different cell-based assays in 384-well plate format.* **a** Mpro-TAGS **b** PLpro-TAGS and **c** FlipGFP-PLproCS transfected HEK293T cells were seeded in 384-well plates before treatment with GC-376 or GRL-0617 for **a, b** 24 h or **c** 48 h, after which nanoLuc intensity level in the medium or fluorescence intensity was quantified in the individual wells. Data are shown as individual data points, n = 16 biological replicates. Z' factor for each assay is indicated above each plot, together with the fold difference in reporter intensity level, calculated by dividing the mean intensity level in the presence of inhibitor by the mean reporter intensity level in the absence of inhibitor. Source data for this figure is provided in a Source Data file.

References

1. Scheller, L. *et al.* Phosphoregulated orthogonal signal transduction in mammalian cells. *Nat. Commun.* **11**, 1–10 (2020).
2. Scheller, L., Strittmatter, T., Fuchs, D., Bojar, D. & Fussenegger, M. Generalized extracellular molecule sensor platform for programming cellular behavior article. *Nat. Chem. Biol.* **14**, 723–729 (2018).
3. Ausländer, D. *et al.* Programmable full-adder computations in communicating three-dimensional cell cultures. *Nat. Methods* **15**, 57–60 (2018).
4. Haellman, V., Strittmatter, T., Bertschi, A., Stücheli, P. & Fussenegger, M. A versatile plasmid architecture for mammalian synthetic biology (VAMSyB). *Metab. Eng.* **66**, 41–50 (2021).
5. Ausländer, S., Fuchs, D., Hürlemann, S., Ausländer, D. & Fussenegger, M. Engineering a ribozyme cleavage-induced split fluorescent aptamer complementation assay. *Nucleic Acids Res.* **44**, 94 (2016).
