# Supplementary material for: Design of modular autoproteolytic gene switches responsive to anti-coronavirus drug candidates
Source: Nat Commun. 2021 Nov 22;12:6786. doi: 10.1038/s41467-021-27072-3 (PMC8609006; doi:10.1038/s41467-021-27072-3)
Supplement: Supplementary file 2 — Reporting Summary [file 41467_2021_27072_MOESM2_ESM.pdf]

nature research

Corresponding author(s): Prof. Dr. Martin Fussenegger

Last updated by author(s): Oct 7, 2021

Reporting Summary

Nature Research wishes to improve the reproducibility of the work that we publish. This form provides structure for consistency and transparency in reporting. For further information on Nature Research policies, see our Editorial Policies and the Editorial Policy Checklist.

Statistics

For all statistical analyses, confirm that the following items are present in the figure legend, table legend, main text, or Methods section.

n/a	Confirmed	
☐	☒	The exact sample size (n) for each experimental group/condition, given as a discrete number and unit of measurement
☐	☒	A statement on whether measurements were taken from distinct samples or whether the same sample was measured repeatedly
☐	☒	The statistical test(s) used AND whether they are one- or two-sided<br>*Only common tests should be described solely by name; describe more complex techniques in the Methods section.*
☒	☐	A description of all covariates tested
☐	☒	A description of any assumptions or corrections, such as tests of normality and adjustment for multiple comparisons
☐	☒	A full description of the statistical parameters including central tendency (e.g. means) or other basic estimates (e.g. regression coefficient) AND variation (e.g. standard deviation) or associated estimates of uncertainty (e.g. confidence intervals)
☐	☒	For null hypothesis testing, the test statistic (e.g. F, t, r) with confidence intervals, effect sizes, degrees of freedom and P value noted<br>*Give P values as exact values whenever suitable.*
☒	☐	For Bayesian analysis, information on the choice of priors and Markov chain Monte Carlo settings
☒	☐	For hierarchical and complex designs, identification of the appropriate level for tests and full reporting of outcomes
☒	☐	Estimates of effect sizes (e.g. Cohen's d, Pearson's r), indicating how they were calculated

Our web collection on statistics for biologists contains articles on many of the points above.

Software and code

Policy information about availability of computer code

Data collection	Luminescence and absorbance data was collected using Tecan Infinite M1000 (TECAN AG, Maennedorf, Switzerland).<br>Microscopic images were collected using microscope Nikon Eclipse Ti
Data analysis	GraphPad Prism 8, ImageJ 1.51

For manuscripts utilizing custom algorithms or software that are central to the research but not yet described in published literature, software must be made available to editors and reviewers. We strongly encourage code deposition in a community repository (e.g. GitHub). See the Nature Research guidelines for submitting code & software for further information.

Data

Policy information about availability of data

All manuscripts must include a data availability statement. This statement should provide the following information, where applicable:

- Accession codes, unique identifiers, or web links for publicly available datasets
- A list of figures that have associated raw data
- A description of any restrictions on data availability

The authors declare that all data supporting the findings of this study are available within the paper and its supplementary information files. All plasmid information is provided in Supplementary Table 3. Requests for materials should be made to the corresponding author. All plasmids generated in this study are available upon request.

Field-specific reporting

Please select the one below that is the best fit for your research. If you are not sure, read the appropriate sections before making your selection.

- [x] Life sciences
- [ ] Behavioural & social sciences
- [ ] Ecological, evolutionary & environmental sciences

For a reference copy of the document with all sections, see nature.com/documents/nr-reporting-summary-flat.pdf

Life sciences study design

All studies must disclose on these points even when the disclosure is negative.

Sample size	No statistical methods were used to predetermine sample size. Sample size was determined based on similar studies in our lab and other published studies in our field (Scheller L, Nat. Commun. 2020; Krawczyk K, Nat. Commun. 2020). n=3 biologically independent samples were predicted to be sufficient for detecting statistically relevant differences between compared groups in cell culture experiments.
Data exclusions	All data was included.
Replication	Attempts at replication were successful, with each experiment replicated at least 3 times. Detailed replication times are listed in each figure legend.
Randomization	For the mouse studies, animals of the same genetic background were randomly allocated into different experimental groups. For cell culture experiments, no covariates based on sample allocations to experimental groups could be observed and no randomization was performed. All direct comparison of induced vs. non-induced were performed with cells transfected under the same conditions with the same transfection mixture.
Blinding	The investigators were not blinded to allocation during experiments and outcome assessment. Blinding was not possible as the same investigator processed the animal/cell culture experiments and analyzed the data.

Reporting for specific materials, systems and methods

We require information from authors about some types of materials, experimental systems and methods used in many studies. Here, indicate whether each material, system or method listed is relevant to your study. If you are not sure if a list item applies to your research, read the appropriate section before selecting a response.

Materials & experimental systems

n/a	Involved in the study	
x		Antibodies
	x	Eukaryotic cell lines
x		Palaeontology and archaeology
	x	Animals and other organisms
x		Human research participants
x		Clinical data
x		Dual use research of concern

Methods

n/a	Involved in the study	
x		ChIP-seq
x		Flow cytometry
x		MRI-based neuroimaging

Eukaryotic cell lines

Policy information about cell lines

Cell line source(s)	Human embryonic kidney cells (HEK293T, ATCC: CRL-11268), adipose tissue-derived human telomerase reverse transcriptase-immortalized human mesenchymal stem cells (hMSC-hTERT, ATCC: SCRC4000), Human fibrosarcoma cel line (HT-1080, ATCC:CCL-121), Golden hamster kidney fibroblasts (BHK-21, ATCC: CCL-10), Human adenocarcinoma cell line (HeLa, ATCC: CCL-2) and Human liver carcinoma cell line (Hep G2, ATCC: HB-8065)
Authentication	Cell lines were authenticated by ATCC and no further authentication was performed, except the phenotype of all cell lines was frequently checked and controlled by microscopy
Mycoplasma contamination	HEK293T cells were tested for mycoplasma and confirmed as negative. Other cell types were not further tested for mycoplasma contamination. We regularly check bacterial contaminations with all routinely used cell lines.
Commonly misidentified lines (See ICLAC register)	Cell lines used in the study are not listed in the database.

Animals and other organisms

Policy information about studies involving animals; ARRIVE guidelines recommended for reporting animal research

Laboratory animals	C57BL6 mice were obtained from Charles River Laboratory, Lyon, France and acclimatized for at least 1 week, kept at 22°C, 50% humidity and 12-h light-dark cycle. Male mice aged 6-8 weeks were used.
Wild animals	The study did not involve wild animals.
Field-collected samples	The study did not involve field-collected samples.
Ethics oversight	All experiments involving animals were performed in accordance with the directives of the European Community Council (2010/63/EU), approved by the French Republic (project no. DR2013-v2).

Note that full information on the approval of the study protocol must also be provided in the manuscript.
